# Circulating miRNA Signatures Associated with Atherosclerosis and Cardiometabolic Comorbidities in People with HIV

**DOI:** 10.3390/medsci14010085

**Published:** 2026-02-12

**Authors:** Marina Martinez-Velasco, José Francisco Sánchez-Herrero, Laura Ibañez, Pablo Velli, Francisco Manuel Muñoz-Lopez, Mireia Cairó, Angeles Jaen, Roser Font, Xavier Martinez-Lacasa, Josep Royo, Joaquim Peraire, Naya Faro-Míguez, Antonio Rivero, Julián Olalla, Pilar Ruiz-Seco, Luis Fernando López-Cortés, Lauro Sumoy, Marta Massanella, David Dalmau

**Affiliations:** 1 Fundació Docència i Recerca Mutua Terrassa, 08221 Terrassa, Spain; 2 IrsiCaixa, 08916 Badalona, Spain; 3 Faculty of Medicine, Universitat de Barcelona, 08036 Terrassa, Spain; 4 High Content Genomics and Bioinformatics Facility, Germans Trias i Pujol Research Institute (IGTP), 08916 Badalona, Spain; 5 Hospital Universitari Mútua de Terrassa, 08221 Terrassa, Spain; 6 Infection and Immunity Research Group (INIM), Institut Investigació Sanitària Pere Virgili (IISPV), 43005 Tarragona, Spain; 7 Hospital Universitari de Tarragona Joan XXIII, 43005 Tarragona, Spain; 8 Medicine and Surgery Department, Universitat Rovira i Virgili (URV), 43003 Tarragona, Spain; 9 Consorcio Centro de Investigación Biomédica en Red de Enfermedades Infecciosas (CIBERINFEC), Instituto de Salud Carlos III, 28029 Madrid, Spain; arivero@uco.es; 10 Hospital Universitario Clínico San Cecilio, 18016 Granada, Spain; 11 Department of Medical and Surgical Sciences, Universidad de Córdoba, 14004 Córdoba, Spain; 12 Hospital Universitario Reina Sofía, 14004 Córdoba, Spain; 13 Instituto Maimónides de Investigación Biomédica de Córdoba (IMIBIC), 14004 Córdoba, Spain; 14 Hospital Universitario Costa del Sol, 29603 Marbella, Spain; 15 Hospital Universitario Infanta Sofía, 28702 San Sebastian de los Reyes, Spain; mprseco@salud.madrid.org; 16 Internal Medicine Department, Universidad Europea de Madrid, 28670 Madrid, Spain; 17 Fundación para la Investigación e Innovación Biomédica del Hospital Universitario Infanta Sofía y Hospital Universitario del Henares (FIIB HUIS-HUHEN), 28702 Madrid, Spain; 18 Hospital Universitario Virgen del Rocío, 41013 Sevilla, Spain; 19 Instituto de Biomedicina de Sevilla (IBiS), 41013 Sevilla, Spain; 20 Consejo Superior de Investigaciones Científicas (CSIC), 41013 Sevilla, Spain; 21 Medicine Department, Universidad de Sevilla, 41004 Sevilla, Spain

**Keywords:** PWH, ART, miRNAs, CVR, atherosclerosis, hypertension, diabetes, obesity, smokers, dyslipidemia, COMVIH-CoR

## Abstract

Background: People with HIV (PWH) experience increased cardiovascular disease driven by chronic inflammation despite suppressive antiretroviral therapy. Circulating microRNAs (miRNAs) have emerged as potential biomarkers of cardiometabolic dysfunction, yet their relevance to HIV-associated atherosclerosis remains unclear. Methods: We analyzed PWH PBMC-derived miRNAs in two independent cohorts: the HUMT cohort (N = 185), characterized by carotid ultrasound assessment of atheroma plaque and carotid intima–media thickness (cIMT), and the CoRIS cohort (N = 119), stratified by cardiometabolic comorbidity burden (≥3 comorbidities vs none). An exploratory miRNA microarray comparing individuals with and without atheroma plaque (AP+ vs. AP−, N = 72) identified candidate miRNAs, a subset of which was selected for validation by RT-qPCR. Associations with atherosclerosis, cardiometabolic comorbidities and the HIV-adapted COMVIH-CoR clinical cardiovascular risk score were examined. Results: Forty-four miRNAs were differentially expressed in AP+ vs. AP– in the microarray. RT-qPCR validation showed sex-specific miRNA association with miR-638 was consistently downregulated in AP+ and pathological cIMT among men, while reduced expression of miR-27b-5p and miR-3613-5p was observed in women. Associations between miRNAs and cardiometabolic comorbidities differed by cohort: in HUMT, miR-638 was reduced in diabetes and obesity, while miR-140-5p and miR-27b-5p were decreased in smokers and individuals with low HDL. CoRIS participants with multiple comorbidities showed a generalized miRNAs upregulation. Notably, miR-140-5p was consistently elevated in individuals with high COMVIH-CoR scores across both cohorts. Conclusions: PBMC-derived miRNAs capture heterogeneous, context-dependent dimensions of cardiovascular risk in PWH, likely reflecting cumulative immune-metabolic stress rather than universal diagnostic markers of subclinical atherosclerosis and supporting a phenotype-specific role.

## 1. Introduction

Antiretroviral therapy (ART) has dramatically reduced the risk of opportunistic infections and mortality associated with severe HIV-related immune dysfunction in people with HIV (PWH) [1]. As a result, PWH are living longer; however, this increased life expectancy has been accompanied by a growing burden of age-related comorbidities that substantially affect long-term survival. Inflammatory pathways activated during HIV infection are not fully normalized by ART, even in individuals with sustained viral suppression [2]. As consequence, persistent immune activation and chronic inflammation play a central role in the development of age-related comorbidities and contribute to features of premature aging in PWH [3].

Cardiovascular disease (CVD) remains a leading cause of morbidity and mortality, with atherosclerosis representing the central pathological process underlying most clinical manifestations [4]. Atheroma plaque formation is a chronic and progressive process characterized by lipid-rich plaque accumulation within the arterial wall, which leads to arterial stiffening and narrowing, impairing blood flow and increasing the risk of ischemic heart disease, stroke, and peripheral artery disease [5]. Carotid intima–media thickness (cIMT) is a widely recognized surrogate marker of subclinical atherosclerosis and is closely associated with both the presence and progression of atheroma plaques [6]. Measured by ultrasound, cIMT quantifies the thickness of the intima and media layers of the carotid artery wall, which reflects early arterial remodeling and provides a non-invasive assessment of vascular disease burden [7].

In PWH, CVD represents a major contributor to morbidity and mortality, with a higher cardiovascular risk than observed in the general population [8]. This excess risk reflects the combined impact of traditional cardiovascular risk factors (i.e., smoking, dyslipidemia, diabetes, and obesity) and HIV-specific mechanisms, particularly chronic inflammation and immune dysregulation and long-term exposure to ART [9,10]. To improve risk estimation in this population, HIV-adapted clinical risk scores such as COMVIH-CoR [11,12], an adaptation of Framingham-based models [13] developed for the Spanish HIV population, have been proposed to estimate 10-year coronary risk. These tools account for the higher risk of coronary events observed in PWH compared with the general population for a given burden of traditional cardiovascular risk factors and are widely used in routine clinical practice due to their simplicity and broad applicability. However, despite improving risk estimation over general-population scores, COMVIH-CoR and similar tools rely primarily on clinical and demographic variables and do not capture the underlying biological mechanisms driving CVD. Moreover, it remains unclear to what extent such clinical risk scores reflect the structural and biological dimensions of cardiovascular risk identified by imaging-based approaches, such as carotid ultrasound, which provide a more direct assessment of subclinical atherosclerosis but require specialized equipment and expertise. This limitation highlights the need for molecular biomarkers that more accurately reflect individual cardiovascular risk.

Circulating microRNAs (miRNAs) are short non-coding ribonucleotide molecules (typically 22 nucleotides) that regulate gene expression across tissues and biological contexts [14]. Owing to their stability, regulated expression, and involvement in key pathological pathways, miRNAs have emerged as promising non-invasive biomarkers for cardiovascular and metabolic diseases [15]. Several miRNAs have been implicated in biological pathways relevant to atherosclerosis and metabolic syndrome, including lipid metabolism, inflammation, glucose homeostasis, and endothelial function [16,17,18].

In PWH, persistent immune activation and metabolic dysregulation despite effective antiretroviral therapy can further reshape miRNA networks, positioning these molecules as potential integrators of immune, metabolic, and vascular pathways relevant to HIV-associated cardiovascular disease [19]. Intracellular miRNAs expressed in peripheral blood mononuclear cells (PBMCs) actively regulate inflammation and endothelial interactions central to vascular injury, offering mechanistic insights beyond plasma miRNAs, as evidenced by recent clinical studies. Whereas circulating miRNAs measured in plasma or serum mainly reflect systemic responses and intercellular communication, miRNAs expressed within PBMCs provide complementary insight into the transcriptional and regulatory status of circulating immune cells, including key HIV targets such as T cells and monocytes [20,21]. In the context of HIV infection, persistent immune dysregulations are well documented within the PBMC compartment [22]. Moreover, as atherosclerosis is largely driven by cells of the monocyte-macrophage lineage [23], PBMC-derived miRNA signatures may link chronic HIV-related immune perturbation with cardiometabolic comorbidities and vascular injury, capturing dimensions of risk not fully reflected by soluble biomarkers or traditional clinical scores. Consistently, whole-blood miRNAs have been linked to vascular function markers [24] and PBMCs-derived related to coronary artery stenosis [25]. Despite this potential, it remains unclear whether miRNAs capture structural vascular disease, cumulative cardiometabolic burden, or clinical risk estimation, and whether these dimensions overlap.

The aim of this study was to characterize PBMC-derived miRNA expression profiles associated with different manifestations of cardiovascular risk in PWH, including subclinical atherosclerosis, cardiometabolic comorbidities, and HIV-adapted cardiovascular risk scores (COMVIH-CoR). We further sought to evaluate the potential utility and limitations of these miRNAs as biomarkers across heterogeneous clinical and biological contexts.

## 2. Materials and Methods

### 2.1. Sample Size and Cohorts

**Figure 1:**
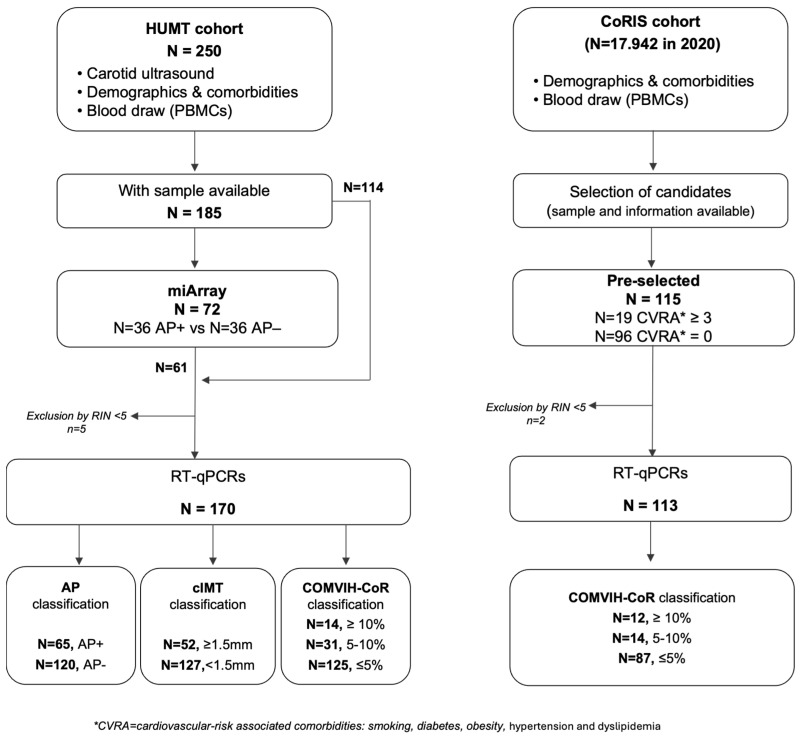
Workflow of sample selection and study design for HUMT and CoRIS cohorts. Flowchart showing the selection of participants included in the exploratory miRNA Array (miArray) and validation phase by RT-qPCR. In the HUMT cohort, carotid artery ultrasound assessment (atheroma plaque status and carotid intima–media thickness [cIMT]), cardiovascular-risk associated comorbidities (CVRA, including smoking, diabetes, obesity, hypertension, and dyslipidemia), and peripheral blood collection for PBMC isolation were all performed during the same clinical visit. Seventy-two HUMT participants were selected for the miArray (N = 36 with atheroma plaque [AP+] and N = 36 without atheroma plaque [AP−]). For RT-qPCRs, 175 HUMT participants were initially considered based on PBMC sample availability. Five samples were excluded due to insufficient RNA quality (RIN ≤ 5), resulting in 170 participants included in the final analyses. In the CoRIS cohort, participants were stratified by CVRA ≥ 3 comorbidities; N = 19 and without any CVRA (0 comorbidities; N = 96). Clinical, demographic, and CVRA corresponded to the same clinical time point as peripheral blood collection. Of the 115 initially selected participants, two samples were excluded due to low RNA integrity (RIN ≤ 5), resulting in 113 individuals included in the final analyses.

A total of 300 PWH were included from two independent cohorts (Figure 1): the cardiovascular cohort from the Hospital Universitari Mútua de Terrassa (HUMT, N = 185) and the Cohort of the Spanish HIV/AIDS Research Network (CoRIS, N = 115).

HUMT cohort. The original HUMT cardiovascular cohort comprised 250 PWH enrolled in 2010 [26]. For the present cross-sectional study, 185 PWH were included based on the availability of both a comprehensive cardiovascular risk assessment (including carotid ultrasound measurements) and stored peripheral blood mononuclear cells (PBMCs). Carotid ultrasound assessment, clinical and cardiovascular-risk associated comorbidities (CVRA), and peripheral blood collection were all performed during the same clinical visit. All individuals were recruited within 6 months (Figure 1).

CoRIS Cohort. CoRIS cohort is an open, prospective, multicenter cohort of adults with confirmed HIV infection, ART-naïve at enrolment. From 2004 to 2020, 17,942 participants have been recruited from 48 centers across 14 of 17 Autonomous Regions in Spain. Data collection follows the HIV Cohorts Data Exchange Protocol (HICDEP) (https://hicdep.org/) and undergoes strict annual quality procedures. The CoRIS database collects baseline and follow-up socio-demographic, immunological, and clinical data, collected according to routine clinical practice. The CoRIS cohort has been previously described in detail [27]. Biological samples were provided by the HIV BioBank. A total of 115 CoRIS participants with available biological samples were included in this study. Participants were selected based on their CVRA. To maximize phenotypic contrast in this study, participants were selected at the extremes of cardiometabolic burden, and intermediate profiles were not included. The high-comorbidity group (N = 19) comprised individuals with ≥3 cardiovascular-related risk factors, including smoking, dyslipidemia, obesity, hypertension, and diabetes. The low-comorbidity group (N = 96) included individuals with no CVRA (Figure 1). For all participants, samples were collected within 6 months of the corresponding clinical, demographic, and comorbidity assessments.

Participants in both cohorts (HUMT and CoRIS) had previously provided written informed consent at the time of enrolment in their respective cohort studies. The HUMT cardiovascular cohort was originally approved by the Ethics Committee of Hospital Universitari Mútua Terrassa (Approval number EO/195; date of approval: 25 January 2017). The CoRIS cohort operates under the approval of the Research Ethics Committee of Hospital General Universitario Gregorio Marañón (Approval number G03/173; date of approval: 1 April 2004), which oversees the national CoRIS protocol.

### 2.2. Cardiovascular Risk Assessments

Carotid artery Ultrasound and Carotid Atheroma Plaque Definition (HUMT cohort). Carotid ultrasonography was performed exclusively in HUMT participants following the recommendations of the Mannheim cIMT Consensus [28]. For each participant, longitudinal B-mode images with a 7 to 14 MHz transductor were obtained from the distal of the common carotid artery on both sides. Participants were examined in the supine position with the head in the midline position and tilted slightly upwards, and images were obtained during cardiac systole. Measurements were taken at both the near and far wall. All ultrasound scans were performed by the same trained operator to minimize variability. Atheroma plaque (AP) was defined according to the Mannheim cIMT Consensus [28], as a focal structure encroaching into the arterial lumen by ≥0.5 mm, or with a thickness ≥ 50% greater than the surrounding IMT, or with an absolute thickness ≥ 1.5 mm. Participants meeting these criteria were classified as AP+.

COMVIH-CoR score (HUMT and CoRIS cohorts). To estimate coronary risk in all study participants, the 10-year coronary risk was calculated using the COMVIH-CoR score, a validated adaptation of the REGICOR/Framingham equation tailored for PWH [11].

### 2.3. Sample Collection, Processing, and Storage

All participants underwent blood drawn into EDTA-coated tubes during the clinical visit. For samples from HUMT cohort, PBMCs were immediately isolated at the site of collection on the same day as blood draw using density-gradient centrifugation with Lymphoprep (Stem Cell, Aix-Shield PoC AS, Oslo, Norway) followed by red blood cell lysing with ACK Lysing Buffer (Lonza, Walkersville, MD, USA). After isolation, cells were washed, counted, resuspended, and cryopreserved in liquid nitrogen until use. For samples from the CoRIS cohort, collected at multiple centers, whole blood was shipped to Madrid the collection day and processed the day after arrival, following the same standard PBMC isolation protocol.

### 2.4. RNA Isolation and Quality Control

PBMC samples were used as the source of intracellular miRNAs. Cryopreserved PBMCs samples were thawed in tempered RPMI media (Hyclone, Logan, UT, USA)) supplemented with 50% Fetal bovine serum. Cells were centrifuged twice at 300 g for 5 min at room temperature and resuspended in 10 mL RPMI 1640 medium supplemented with 10% of FBS. Cells were mixed 1:1 with 0.4% trypan blue solution, and total and viable cell numbers were determined using a Neubauer hemocytometer by trypan blue exclusion. Total RNA, including the small RNA fraction, was extracted from PBMCs using the mirVana™ miRNA Isolation Kit with phenol (Invitrogen, Carlsbad, CA, USA; Thermo Fisher Scientific, Waltham, MA, USA), which is optimized for the recovery of miRNAs and other small RNAs, following manufacturer’s instructions. RNA concentration was assessed with a Nanodrop 2000 (Thermo Fisher Scientific). Samples with <117 ng/µL of RNA were pre-amplified to obtain a minimum of 350 ng of total RNA using Custom Reverse Transcription Pools and Custom Preamplification Pools with TaqMan^®^ MicroRNA Assays (Thermo Fisher Scientific) following manufacturer’s instructions. RNA integrity was evaluated using the Bioanalyzer 2100 (Agilent Tech, Santa Clara, CA, USA) with the Agilent RNA 6000 Nano kit, along with the Agilent 6000 Pico kit (Agilent Tech) [29]. Samples with an RNA Integrity Number (RIN) ≤ 5 were excluded (N = 7), RNA degradation can impair extraction yield and reverse transcription efficiency [29].

### 2.5. Exploratory miRNA Microarray (HUMT Cohort)

An exploratory miRNA Array analysis was conducted using 72 samples from the HUMT cohort, selected to compare PWH with atheroma plaque (AP+, N = 36) and those without plaque (AP−, N = 36). All selected samples had a RIN > 7. Microarray profiling was performed using the Human GeneChip™ miRNA 4.1 Assay (Applied Biosystems™, Foster City, CA, USA) at the IDIBAPS Genomics Service Unit (Barcelona, Spain). Bioinformatic analysis and preprocessing of the raw data information was performed in the High Content Genomics and Bioinformatics Unit at Germans Trias i Pujol Research Institute (IGTP).

### 2.6. Validation of Differentially Expressed miRNA by RT-qPCR Assays

To confirm the miRNAs differentially expressed in the miArray, we performed reverse transcription quantitative PCR (RT-qPCR) assays including 283 of the 300 samples from the HUMT (N = 170) and CoRIS (N = 113) cohorts, selected based on biological sample availability and adequate RNA integrity (RIN > 5) (Figure 1). A two-step RT-qPCR was performed to quantify selected miRNAs: miR-140-5p (#001187), miR-146b-5p (#001097), miR-27b-5p (#002174), miR-3613-5p (#463197), miR-4668-3p (#463182), and miR-638 (#001582), all from Thermo Fisher Scientific. cDNA synthesis was performed using the miRNA Reverse Transcription kit (Thermo Fisher Scientific), and quantification was carried out using TaqMan Fast Advanced Master Mix technology (Thermo Fisher Scientific). RNU44 (#001094, Thermo Fisher Sci) and RNU48 (#001006, Thermo Fisher Scientific) were used as endogenous controls. Target miRNAs were detected with FAM-labelled probes, and endogenous controls (RNU44, RNU48) with VIC-labelled probes (TaqMan, Thermo Fisher Scientific). Negative RT controls were included to exclude non-specific amplification or genomic RNA contamination. All qPCR reactions were run on the 9700HT Fast Real-Time PCR System (Applied Biosystems, Thermo Fisher Scientific). Relative miRNA expression levels were calculated using the 2^−ΔCt^ method, normalizing target miRNA Ct values to the geometric mean of the endogenous controls RNU44 and RNU48. These normalized expression values were used for all subsequent statistical analyses. We observed significant differences in endogenous control miRNA Ct values (RNU44 and RNU48) between cohorts, likely related to differences in PBMC handling, and RT-qPCR data were therefore analyzed separately (Supplementary Figure S1).

### 2.7. Statistical Analysis

Comparisons of demographic and clinical variables between groups were performed using the Mann–Whitney U test for continuous variables and Fisher’s exact test for categorical variables. For microarray analysis, Oligo R package (version 1.46.0) [30] was used to read Affymetrix CEL files along the annotation package [31] specifically designed for the miRNA Affymetrix GeneChip 4.1. To remove non-specific binding or spatial heterogeneity across the array and normalize between samples, background was corrected and normalized by robust multichip average (RMA). Data were plotted after dimension reduction to assess similarity patterns among samples, to identify possible outlier samples and/or batch effects that might be confounding the effect of the conditions of interest, performing two different reduction techniques (Principal Components Analysis—PCA, Multi-Dimensional Scaling—MDS). We used limma [32] to generate a DE analysis for data normalized using RMA normalization and cutoff values for absolute fold change |FC| > 1.2 and for nominal *p*-value < 0.05. Given the exploratory nature of the microarray analysis, nominal *p*-values combined with effect size thresholds were used to prioritize candidate miRNAs for downstream RT-qPCR analyses. Multiple testing correction was therefore not applied. Functional analyses were later performed for miRNA candidates. Firstly, we used the miRTarBase [33], to retrieve experimentally validated miRNA–target interactions and then we analyzed the enriched gene sets using Gene Set Enrichment Analysis (GSEA) via the FGSEA [34] R package against the MSigDB collections [35]. We also retrieved information from the RNA central repository by EMBL-EBI, miRNA Enrichment Analysis and Annotation Tool (miEAA [36] TAM 2.0 (Tool for miRNA Set Analysis [37]), and Human miRNA Disease Database [38].

For RT-qPCR data, non-parametric comparisons between two groups were performed using the Mann–Whitney U test, while comparisons across more than two groups were assessed using the Kruskal–Wallis test with Dunn’s post hoc correction for multiple pairwise comparisons. Correlation and multivariable regression analyses were interpreted in an exploratory manner without global adjustment for multiple testing. Correlations between variables was assessed by Spearman’s rank correlation coefficient. Heatmaps were generated in R (version 4.5.2; R Foundation for Statistical Computing, Vienna, Austria) using the “ComplexHeatmap” package (version 2.26.0) after scaling each miRNA to zero mean and unit variance across individuals and plotting the median expression per across the relevant clinical groupings to represent miRNA expression patterns across samples. The Venn diagrams were generated using the interactiVenn web application (http://www.interactivenn.net, accessed on 11 January 2026) and subsequently edited for final presentation with the vector graphic software Inkscape (version 1.4.2 The Inkscape Project). For multivariate analysis, normality was checked via Q-Q plots and transformations were applied to each miRNA. Linear regression models were fitted for each miRNA against covariates (smoking, obesity, hypertension, diabetes, dyslipidemia, cholesterol, HDL) using R (version 4.5.2; R Foundation for Statistical Computing).

## 3. Results

### 3.1. miRNA Discovery Identifies an Atherosclerosis-Associated Signature

**Figure 2:**
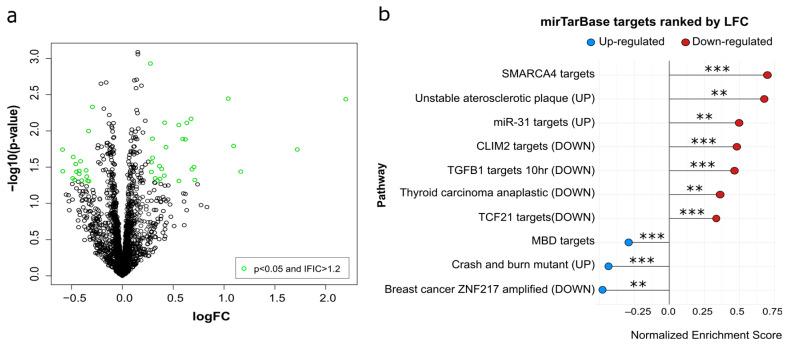
Differential miRNA expression among miArray and pathway enrichment analysis in miRNAs detected. (**a**) Volcano plot showing the distribution of miRNAs according to log fold change (logFC) and statistical significance when comparing AP+ and AP− individuals. miRNAs meeting significance thresholds (unadjusted *p* value < 0.05 and |logFC| > 1.2) are highlighted in green. (**b**) mirTarBase pathway enrichment analysis ranked by logFC (LFC). Color indicates whether the pathway enriched is up-regulated or down-regulated. Statistical significance is indicated as follows: *p* < 0.01 (**), and *p* < 0.001 (***).

To identify circulating miRNAs associated with subclinical atherosclerosis in PWH, we first performed a microarray analysis in a subset of participants from the HUMT cohort classified according to the presence (AP+, N = 36) or absence (AP−, N = 36) of carotid atheroma plaque. The clinical and demographic characteristics of the microarray subset were comparable to those of the overall HUMT cohort and are shown in Supplementary Table S1). A total of 44 miRNAs were differentially expressed between groups, meeting both statistical significance (unadjusted *p*-value < 0.05) and effect size criteria (|log_2_FC| > 1.2) (Figure 2a and Supplementary Table S2).

To explore the biological relevance of the miRNAs identified, we retrieved experimentally validated miRNA–target interactions using mirTarBase and performed a gene set enrichment analysis of genes against the MSigDB collections. This analysis revealed a significant enrichment within the C2_CGP curated gene sets, including a signature capturing genes characteristically upregulated in human unstable atheroma plaques (*p* = 0.0012, Figure 2b). This enrichment was driven by targets of miRNAs that were downregulated (in red) in AP+ individuals versus AP−, suggesting that reduced circulating miRNA levels may release post-transcriptional repression of genes previously described as upregulated (UP in Figure 2b) in unstable plaques.

Based on these results, six miRNAs were selected for validation. Three miRNAs (miR-140-5p, miR-3613-5p and miR-638) were selected based on reported involvement in lipid metabolism, statin response and vascular biology, respectively. miR-4668-3p was included as an exploratory candidate owing to the absence of prior functional or mechanistic information. In addition, two miRNAs (miR-146b-5p and miR-27b-5p) were included based on previous associations in HIV studies (30), despite not being differentially expressed in the microarray analysis. All six miRNAs were initially evaluated by RT-qPCR; however, miR-4668-3p was excluded from subsequent analyses due to suboptimal amplification efficiency.

### 3.2. Validation of Candidate miRNAs in the HUMT Cohort

A total of 170 HUMT participants were included in the validation analyses after accounting for biological sample availability and RNA quality. Clinical and demographic characteristics of the HUMT cohort stratified by atheroma plaque status are shown in Table 1.

Individuals with atheroma plaque (AP+) were older than those without (AP−) (*p* < 0.0001) and had a longer duration since HIV diagnosis (*p* = 0.01). Men represented 77% of the cohort, with no differences between groups. AP+ individuals also exhibited higher cIMT (*p* < 0.0001), slightly higher COMVIH-CoR scores (*p* = 0.0526) and a more atherogenic lipid profile, characterized by increased levels of total cholesterol (*p* = 0.0027), LDL cholesterol (*p* = 0.0119) and triglycerides (*p* = 0.0042). Obesity was more frequent among AP+ individuals (*p* = 0.0143), although median body mass index did not differ significantly between groups.

**Figure 3:**
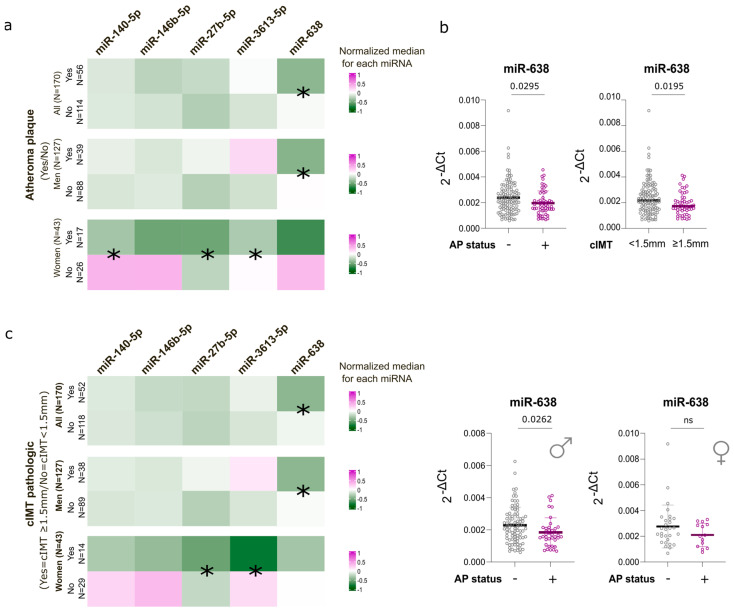
Case–control comparison of circulating miRNAs according to atheroma plaque presence and cIMT status (cIMTs ≥1.5 mm were considered cardiovascularly pathologic) in the HUMT cohort. (**a**) Heatmap showing expression of miRNAs in individuals with (AP+) and without (AP−) atheroma plaque. (**b**) Individual miR-638 expression values in HUMT participants stratified by AP status and cIMT category, shown for the overall cohort and after stratification by sex. (**c**) Heatmap showing expression of miRNAs in participants with cIMT pathologic (≥1.5 mm) and non-pathologic (<1.5 mm). For both heatmaps, values were normalized within the color scale, and medians are displayed in the heatmap. Mann–Whitney test has been applied in comparisons among groups. Significant differences (unadjusted *p* < 0.05) between groups are indicated (*). ns: not significant.

RT-qPCR analyses confirmed differential expression of several candidate miRNAs according to AP status (Figure 3a). When analyzing all the cohort together, miR-638 was the only miRNA significantly downregulated in AP+ individuals compared with AP-participants (*p* = 0.03, Figure 3a). Given known biological differences between men and women in cardiovascular disease, analyses were further stratified by sex. Sex-stratified analyses were performed for descriptive purposes; formal interaction testing was not powered, and findings should therefore be considered exploratory. In men, miR-638 remained significantly downregulated in AP+ individuals (*p* = 0.03), whereas this association was not observed in women (Figure 3a,b). In contrast, among women, AP+ status was associated with lower expression of miR-140-5p (*p* = 0.04), miR-27b-5p (*p* = 0.02), and miR-3613-5p (*p* = 0.009). However, these results should be interpreted with caution due to the limited sample size among women (N = 17 AP+ and N = 31 AP−).

To further assess whether miR-638 was associated with structural characteristics of atherosclerosis beyond plaque presence, HUMT participants were additionally stratified according to cIMT. A cIMT threshold of 1.5 mm was used to define pathological arterial thickening (cIMT < 1.5 mm the non-pathologic group and cIMT ≥ 1.5 mm as pathologic), which in this cohort corresponded to a subset of participants with atheroma plaque and reflects more advanced plaque-related remodeling. Using this stratification, miR-638 expression followed the same pattern observed in the plaque-based analyses, with significantly lower levels in individuals with cIMT ≥ 1.5 mm compared to those with cIMT < 1.5 mm (*p* = 0.02) (Figure 3b,c). This association was present in men but not in women (Figure 3c). Similar to the AP stratification, pathological cIMT in women was associated with lower expression of miR-27b-5p (*p* = 0.02) and miR-3613-5p (*p* = 0.009) (Figure 3c).

To evaluate whether miRNA expression levels were associated with arterial thickness across the full spectrum of disease severity, we assessed Spearman correlations between miRNA expression levels and continuous cIMT values. No significant correlations were observed between any of the miRNAs and cIMT values, and this lack of association remained unchanged when analyses were stratified by sex (Supplementary Table S3).

### 3.3. miRNA Expression According to Cardiovascular-Related Comorbidities

To explore the clinical relevance of these miRNAs beyond imaging-defined atherosclerosis, we next assessed miRNA expression according to individual cardiometabolic comorbidities, including smoking, diabetes, hypertension, obesity, dyslipidemia, and HDL cholesterol levels. Analyses were performed separately in the HUMT and CoRIS cohorts due to differences in cohort structure and sample processing.

Within the HUMT cohort, several miRNAs showed differential expression across individual comorbidities (Supplementary Figure S2). miR-140-5p and miR-27b-5p were downregulated in active smokers (*p* = 0.007 and *p* = 0.005, respectively) and in individuals with low HDL cholesterol (≤40 mg/dL; *p* = 0.028 and *p* = 0.0157). miR-27b-5p was upregulated in participants with hypertension (*p* = 0.0183), while miR-638 was downregulated in individuals with diabetes (*p* = 0.0093) and obesity (*p* = 0.022).

In CoRIS cohort, cardiovascular risk was defined according to comorbidity burden, comparing individuals with ≥3 cardiovascular-risk associated comorbidities (CVRA ≥ 3) to those without (CVRA = 0) (Table 2). Age did not differ significantly between groups (*p* = 0.28). As expected from the cohort design, marked differences were observed across classical cardiovascular risk factors, including smoking (*p* < 0.0001), BMI and obesity (*p* < 0.0001), dyslipidemia (*p* < 0.0001), hypertension (*p* < 0.0001), and type 2 diabetes (*p* < 0.0001). The CVRA ≥ 3 group also showed higher triglyceride levels (*p* = 0.0002) and lower HDL levels (*p*-value = 0.058). Men represented 95% of the CoRIS cohort. Given extreme cardiometabolic phenotyping, contrasting individuals with no comorbidities against those with ≥3 simultaneous comorbidities, miRNA expression likely reflects the combined impact of multiple cardiometabolic stressors and differed markedly from those observed in the HUMT cohort (Supplementary Figure S2). In this context, all five miRNAs were upregulated in participants with diabetes: miR-140-5p (*p* = 0.0168), miR-146b-5p (*p* = 0.0012), miR-27b-5p (*p* = 0.0225), miR-3613-5p (*p* = 0.0037), and miR-638 (*p* = 0.0003). Four out of 5 miRNAs were also upregulated in obese participants compared to no-obese: miR-140-5p (*p* = 0.0168), miR-146b-5p (*p* = 0.0148), miR-3613-5p (*p* = 0.0346), and miR-638 (*p* = 0.0464). Similarly, miR-146b-5p (*p* = 0.0071) and miR-638 (*p*-value = 0.0344) were also upregulated in hypertensive participants.

### 3.4. miRNAs and Clinical Cardiovascular Risk Estimation (COMVIH-CoR)

**Figure 4:**
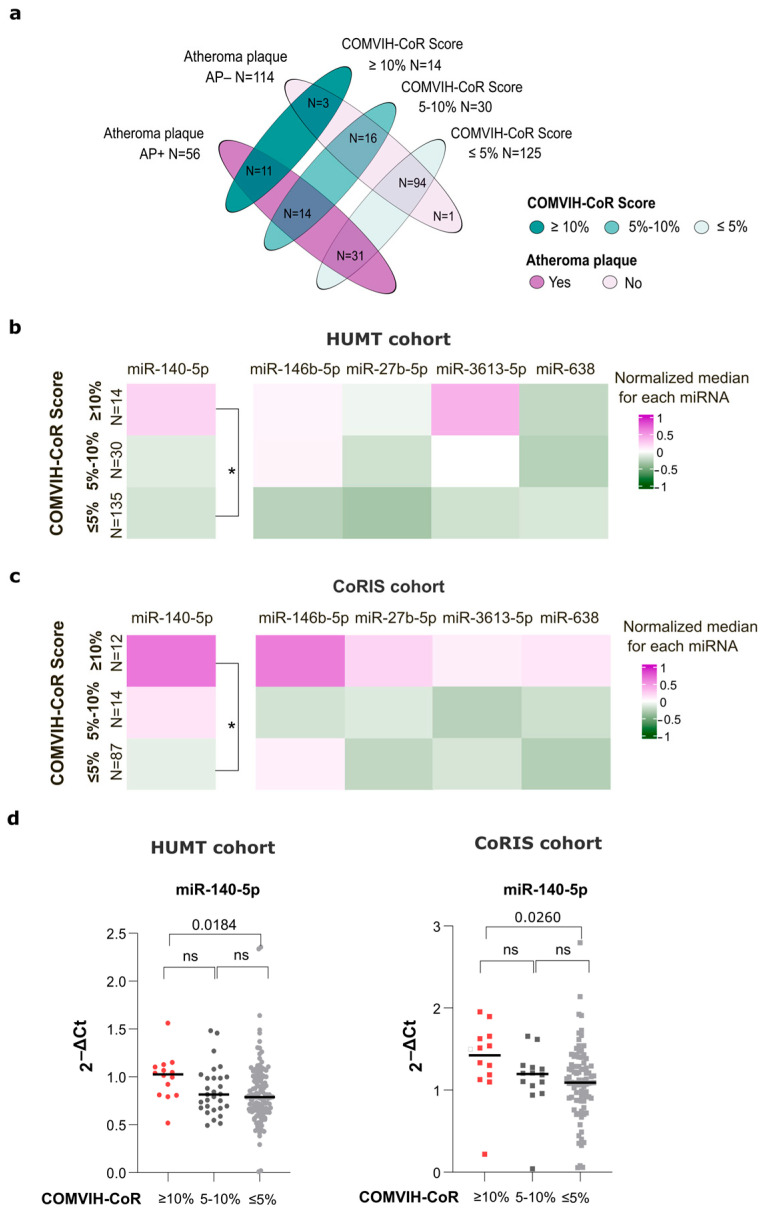
Association between COMVIH-CoR Score and miRNAs expression. (**a**) Venn diagram showing the distribution of participants according to atheroma plaque status (presence [AP+] or absence [AP−]) and COMVIH-CoR score categories (low ≤ 5%, intermediate 5–10%, and high risk ≥ 10%). Numbers indicate the count of individuals meeting each combination criteria. Colors denote atheroma plaque status (purple = AP+, light purple = AP−) and COMVIH-CoR score categories (teal = ≥10%, medium teal= 5–10%, 10% and light teal = ≤5%). Heat map of miRNA expression in HUMT (**b**) and CoRIS (**c**) participants stratified by COMVIH-CoR score. Values were normalized within the color scale, and medians are displayed in the heatmap. Kruskall–Wallis comparisons were performed with Dunn’s post hoc correction for multiple pairwise comparisons. Significant differences (unadjusted *p* < 0.05) between groups are indicated (*). (**d**) Individual expression values of miR-140-5p according to COMVIH-CoR score categores in HUMT and CoRIS cohorts. ns: not significant.

To contextualize the RT-qPCR analyses, we first examined the overlap between the two cardiovascular risk classifications used in the HUMT cohort: the presence of atheroma plaque and the COMVIH-CoR score, grouped as low risk (≤5%), intermediate risk (5–10%), and high risk (≥10%). As shown in Figure 4a, these classifications only partially overlapped. Among the 170 HUMT participants included in the RT-qPCR analyses, 114 showed no detectable atheroma plaque (AP−) and 56 were classified as AP+. Based on COMVIH-CoR score, 125 participants were categorized as low risk (≤5%) and 14 as high risk (≥10%), and 30 individuals fell into the intermediate-risk category. Notably, several AP+ individuals (N = 31) were classified as low risk by COMVIH-Cor, while some individuals with high COMVIH-CoR risk group did not present atheroma plaque (N = 3). Only a limited subset of participants met both high-risk definitions (N = 11), indicating that plaque-based imaging and clinical risk scores capture distinct, albeit partially overlapping, dimensions of cardiovascular risk in PWH. Similar distributions were observed after stratification by sex (Supplementary Figure S3). Clinical characteristics of HUMT individuals stratified by COMVIH-CoR score are provided in Supplementary Table S4.

Given this limited overlap between structural atherosclerosis and calculated cardiovascular risk, we next examined whether miRNA expression differed across COMVIH-CoR score categories. Because COMVIH-CoR was available for both HUMT and CoRIS participants, this analysis enabled a harmonized assessment of clinical cardiovascular risk across cohorts. In this context, miR-140-5p expression was significantly higher in the high-risk group compared with the low-risk one in both cohorts (*p* = 0.0184 and *p* = 0.0260 in HUMT and CoRIS, respectively, Figure 4b–d). No other miRNAs showed significant differences across COMVIH-CoR categories. Sex-stratified analyses were not performed for COMVIH-CoR due to the low number of women, particularly in the high-risk category, which precluded adequately powered comparisons.

Finally, because several clinical variables contributing to COMVIH-CoR are continuous (including age and lipid parameters), we assessed correlations between miRNA expression levels and these variables, as well as with the COMVIH-CoR score itself. Although some associations reached nominal statistical significance (*p* < 0.05), the correlations coefficients were consistently low (Spearman’s r < 0.25), indicating weak relationships between miRNA expression and continuous cardiovascular risk measures (Supplementary Table S5).

To further evaluate whether combinations of traditional cardiovascular risk factors jointly influenced miRNA expression, we next performed multivariable linear regression analyses (Supplementary Table S6). Overall, these models explained only a modest proportion of the variance in circulating miRNA levels in both cohorts, with adjusted R^2^ values remaining low (up to 0.09 for miR-27b 5p). In the HUMT cohort, miR-27b 5p exhibited the clearest associations with clinical variables, with significantly lower levels in smokers and higher levels in hypertensive individuals, while miR-638 was inversely associated with both obesity and diabetes and no other covariates showed consistent significant effects. Within CoRIS cohort, only miR-638 showed an association, being downregulated in smoking participants (Supplementary Table S6).

## 4. Discussion

In this study, we demonstrate that PBMC-derived miRNA expression profiles capture distinct and complementary dimensions of cardiovascular risk in PWH, integrating imaging-defined subclinical atherosclerosis, clinical risk estimation, and cardiometabolic comorbidities. By combining vascular imaging, the HIV-adapted COMVIH-CoR score, and comorbidity-based phenotyping across two independent cohorts, our findings highlight the context-dependent nature of miRNA regulation in HIV-associated CVD.

The most consistent imaging-associated signal in the HUMT cohort was the downregulation of miR-638 in individuals with carotid atheroma plaque and in those meeting the pathological cIMT threshold (≥1.5 mm). Although atheroma plaque presence and increased cIMT are related manifestations of subclinical atherosclerosis, they capture partially distinct biological processes [39]. The association of miR-638 with both plaque presence and advanced arterial thickening suggests that this miRNA may reflect plaque-related structural remodeling rather than plaque presence alone. These findings are concordant with a previous study showing reduced miR-638 expression in symptomatic compared with asymptomatic carotid atherosclerotic disease, supporting a link with more advanced or unstable vascular phenotype [40]. From a mechanistic perspective, experimental studies have implicated miR-638 in pathways regulating cellular metabolism and proliferation, including repression of phosphoglycerate kinase 1 (PGK1) and modulation of downstream mTOR signaling, which in experimental endothelial models promotes endothelial cell proliferation and survival [41]. Dysregulation of this pathway has been implicated in disrupting vascular tone, barrier integrity, and anti-inflammatory signaling, supporting a potential role of miR-638 in neointimal formation and vascular wall remodeling [42]. However, as our analyses were performed in PBMCs, these observations should be interpreted as reflecting systemic immune–metabolic alterations associated with advanced subclinical atherosclerosis rather than direct effects on vascular structure. Functional studies in immune and vascular cells from PWH will be required to establish causality.

Beyond overall associations, we identified marked sex-specific miRNA expression patterns across imaging-based stratifications. The association between miR-638 and plaque or cIMT severity was most evident in men, whereas among women, miR-27b-5p and miR-3613-5p were consistently downregulated in both atheroma plaque-positive individuals and those with pathological cIMT. However, given the limited number of women included in the present study, these sex-specific associations should be interpreted as exploratory, requiring confirmation in larger cohorts. Sex differences in miRNA expression and atherosclerosis biology likely arise from both genetic and hormonal factors. The X chromosome harbors the highest density of annotated miRNA genes, which may contribute to sex-biased miRNA expression patterns [43]. In parallel, sex differences in atherosclerosis phenotype have been described, with men tending to develop larger and more calcified atheroma plaques, whereas women exhibit more frequently stable lesions [44]. Together, these observations support the relevance of sex-stratified analyses when evaluating miRNA signatures associated with subclinical atherosclerosis in PWH.

miR-27b-5p regulated immune metabolic pathways relevant to atherosclerosis, particularly trough PPARγ, a master regulator of lipid storage and adipogenesis [45]. In this context, the consistent downregulation of miR-27b-5p in women with atheroma plaque and pathological cIMT may reflect sex-specific alterations in immune–metabolic regulation associated with subclinical atherosclerosis, although causal mechanisms remain to be established.

In contrast, miR-3613 remains poorly characterized in cardiovascular disease. Existing studies have primarily linked miR-3613 to cell cycle regulation [46,47,48,49] and endometriosis [47,50]. Based on our results, the downregulation of miR-3613-5p in women with plaque and increased cIMT raises the hypothesis that this miRNA may be related to dysregulated cell cycle control or proliferative responses in vascular or immune cells [46] However, this interpretation remains speculative, and functional studies will be required to clarify the biological role of miR-3613-5p in sex-specific atherosclerotic processes.

Given the multifactorial nature of cardiovascular risk in PWH, we next examined whether the miRNAs identified in imaging-based analyses were also associated with major comorbidities. This approach allowed us to assess whether miRNA regulation extended beyond structural arterial changes and reflected broader metabolic and inflammatory burden. Within the HUMT cohort, miR-638 was also downregulated in individuals with diabetes and obesity. A previous study has linked miR-638 to early dysglycemia, supporting a potential role in glucose metabolism [51]. However, in our cohort, the number of diabetic participants was limited (N = 5), and most of them (75%) presented an atheroma plaque, making it difficult to disentangle metabolic effects from underlying vascular disease. Similarly, the majority of obese participants exhibited atheroma plaque, suggesting that the observed miR-638 downregulation may primarily reflect advanced subclinical atherosclerosis rather than obesity per se.

In contrast to miR-638, miR-140-5p and miR-27b-5p showed consistent associations with smoking and low HDL cholesterol levels in HUMT participants, suggesting regulation driven by shared lipid and inflammatory perturbations rather than by plaque-specific vascular remodeling. Both miRNAs are involved in lipid handling and macrophage biology, processes that are highly sensitive to systemic metabolic stress. miR-27b-5p is a key regulator of cholesterol efflux and macrophage lipid handling and is sensitive to stimuli that disrupt lipid homeostasis [45]. miR-140-5p has been related also to lipid metabolism and macrophage function, although published data report context-dependent and sometimes opposing effects [52,53]. Beyond intracellular regulation, circulating miRNA levels may also be influenced by lipoprotein-mediated transport, as several miRNAs are transported by HDL particles [54]. Accordingly, reduced HDL levels may directly affect the abundance and stability of miR-140-5p and miR-27b-5p in circulation. In parallel, cigarette smoke exposure induces oxidative stress and suppresses transcriptional programs involved in lipid efflux, repressing the expression of several miRNAs [55]. Consistent with this mechanism, long-term cigarette smoke exposure has been shown to downregulate both miR-27b and miR-140-5p [56], closely aligning with the associations observed in our cohort.

Notably, miR-27b-5p displayed divergent behavior across comorbidities, being downregulated in smokers and individuals with low HDL, yet upregulated in participants with hypertension. One possible mechanistic framework described in the literature links miR-27b-5p to repression of the plasma membrane calcium-transporting ATPase 1 (ATP2B1) [57], key regulator of intracellular calcium homeostasis in vascular smooth muscle and endothelial cells [58]. Mouse models with specific knockout of ATP2B1 develop elevated blood pressure and increased intracellular Ca^2+^ in vascular smooth muscle, supporting a role for this pathway in hypertension. In this context, the upregulation of miR-27b-5p in hypertensive individuals may be consistent with altered calcium-related stress responses. However, because ATP2B1 expression and calcium signaling were not directly assessed in the present study, this interpretation remains hypothetical and requires targeted functional validation.

In the CoRIS cohort, miRNA expression patterns differed markedly from those observed in the HUMT cohort, reflecting distinct dominant biological contexts rather than cohort-specific discrepancies. Whereas miRNA associations in the HUMT cohort were primarily linked to imaging-defined subclinical atherosclerosis, miRNA regulation in the CoRIS cohort appeared to reflect cumulative cardiometabolic stress arising from the coexistence of multiple risk factors. This cohort was intentionally enriched for individuals at opposite extremes of cardiometabolic burden, contrasting participants without cardiovascular-related comorbidities with those presenting multiple concurrent conditions. Under this extreme phenotypic contrast, miRNA expression appeared to reflect cumulative cardiometabolic stress rather than isolated risk factors or structural vascular alterations. In this context, diabetic and obese participants showed a generalized upregulation of most analyzed miRNAs, with additional increases observed in hypertensive individuals (miR-146b-5p and miR-638). Among individuals with diabetes, 16% were also obese or hypertensive, and 32% presented both obesity and hypertension. The extensive coexistence of cardiometabolic comorbidities within this high-risk subgroup suggests that miRNA dysregulation in CoRIS likely arises from the combined impact of chronic inflammation, metabolic stress, and endothelial dysfunction, rather than from any single clinical condition. This pattern contrasts with the more heterogeneous HUMT cohort, where individual comorbidities could be examined separately and showed more selective miRNA associations.

miR-146b-5p exemplifies this pattern, as it is a well-established anti-inflammatory miRNA, and its expression is induced under conditions of sustained low-grade inflammation, where it acts as a negative regulator of NF-Κb and TLR-4 signaling to limit tissue damage [59]. Its consistent upregulation in individuals with high cardiometabolic burden therefore likely reflects chronic immune activation rather than structural atherosclerosis. Similarly, the upregulation of miR-27b-5p and miR-140-5p in diabetic and obese individuals may represent stress-responsive adaptations to metabolic and inflammatory signaling rather than direct markers of atherosclerotic burden.

miR-638 displayed different expression patterns across cohorts, being overexpressed in diabetic, hypertensive, and obese participants in CoRIS, but downregulated in individuals with atheroma plaque in HUMT. Given the substantial biological and phenotypic differences between both cohorts, divergent miRNA expression patterns are not unexpected and likely reflect different dominant biological pressures rather than contradictory biology. However, the mechanisms underlying miR-638 regulation across distinct cardiometabolic and vascular contexts remain incompletely understood and warrant further investigation.

In contrast to miR-638, miR-140-5p was not consistently associated with imaging-defined subclinical atherosclerosis but instead emerged as a marker of clinical cardiovascular risk and cardiometabolic stress. Its upregulation in individuals with elevated COMVIH-CoR scores across both cohorts, together with its associations with smoking and low HDL cholesterol, suggests that miR-140-5p may reflect systemic inflammatory–metabolic burden rather than plaque-specific vascular remodeling. This interpretation is consistent with previous reports describing miR-140-5p as a stress-responsive miRNA regulated by oxidative and inflammatory signaling pathways [53,60]. Within this framework, miR-140-5p may capture adverse cardiovascular risk profiles in PWH, complementing imaging-based markers that primarily reflect structural arterial disease.

Several study limitations should be acknowledged. First, the exploratory microarray analysis did not identify a single miRNA with a large effect size when comparing individuals with and without atheroma plaque, reflecting the biological heterogeneity of subclinical atherosclerosis in PWH. To address this, we combined pathway-level analyses, targeted validation, and implemented multiple complementary cardiovascular phenotypes to strengthen biological interpretation. Second, the HUMT and CoRIS cohorts could not be combined for joint analyses due to differences in sample processing and PBMC isolation timelines, which resulted in batch effects (Supplementary Figure S1). As a result, all analyses were performed in a cohort-specific manner, which may affect absolute miRNA expression levels and limit direct quantitative comparisons between cohorts. However, this approach allowed us to examine miRNA regulation within well-defined clinical and biological contexts, highlighting context-dependent patterns rather than technical artefacts alone. Third, in the CoRIS cohort, individuals were selected from the extremes of comorbidity burden to maximize biological contrast and improve signal detection. While this strategy strengthens internal validity, it limits assessment of dose–response relationships and may reduce generalizability to intermediate-risk phenotypes. Fourth, HCV co-infection was not included as a covariate in the miRNA models due to its very low prevalence in the CoRIS cohort and its balanced distribution across atheroma plaque groups in the HUMT cohort; therefore, a potential contribution of HCV co-infection to inter-cohort differences in miRNA expression cannot be fully excluded. Finally, sex-stratified analyses, particularly in the CoRIS cohort, were limited by the lower number of women, and sex-specific findings should be considered hypothesis-generating. Additional limitations include the cross-sectional design, which precludes causal inference, and the use of PBMC-derived miRNAs, which capture systemic immune-metabolic alterations rather than vascular tissue–specific processes. Despite these limitations, the consistency of key findings across phenotypes and cohorts supports the robustness of our conclusions.

Collectively, our findings highlight pronounced context-dependence in miRNA expression across cardiovascular phenotypes. Importantly, the divergent miRNA expression patterns observed between cohorts should not be interpreted as cohort-specific discrepancies, but rather as reflecting distinct biological contexts. In the HUMT cohort, we observe associations with imaging-defined atherosclerosis, whereas in the CoRIS cohort, miRNA expression appears to be related to cumulative cardiometabolic stress. Similar variability has been reported in other settings, where miRNA expression patterns differ according to tissue type, disease stage, age, and clinical context, and may be associated with divergent prognostic implications [61]. This heterogeneity underscores the limitations of miRNAs as robust, universal biomarkers for subclinical atherosclerosis and overall cardiovascular risk. In this context, these miRNAs may be informative as indicators of immune-metabolic stress and serve as complementary readouts alongside vascular imaging and/or clinical risk scores, rather than standalone diagnostic biomarkers. Instead, miRNAs may offer phenotype-specific insights into distinct biological processes underlying HIV-associated CVD. Although this study was conducted in PWH, miRNA expression patterns appeared to be more closely associated with cardiometabolic and immune-related comorbidity burden than with classical HIV-specific clinical markers. Associations with CD4 cell counts, nadir CD4, and virological parameters were generally weak under suppressive ART (Table S7), suggesting that miRNA regulation in this context may primarily reflect chronic immune–metabolic stress rather than ongoing HIV replication. These findings support a context-dependent model of miRNA regulation in long-term treated HIV infection. Future studies integrating functional validation, longitudinal follow-up, and adequately powered sex-stratified analyses will be essential to clarify their mechanistic roles and translational relevance of these miRNAs. From a translational perspective, these findings argue against single-miRNA diagnostics but support their integration into multi-dimensional cardiovascular risk models.

Taken together, our findings indicate that PBMC-derived miRNAs are associated with specific cardiovascular phenotypes and inflammatory-metabolic states in PWH, rather than representing universal markers. These observations further support the notion that PBMC-derived miRNAs respond differentially depending on the dominant clinical context, such as imaging-defined subclinical atherosclerosis or cumulative cardiometabolic stress. While these miRNAs capture biologically relevant dimensions of cardiovascular risk, their predictive value for vascular progression or future cardiovascular events remains to be determined in studies with longitudinal molecular profiling.

## Figures and Tables

**Table 1:** Clinical, demographic, and cardiovascular characteristics of participants from HUMT cohort stratified by atheroma plaque presence (AP+) or absence (AP−).

	AP+ N = 56	AP− N = 114	*p*-Values
Age, years, median (IQR)	49 (45–52)	42 (38–47)	<0.0001
Sex, Male, N (%)	39 (70)	88 (77)	0.3483
Ethnicity, N (%)			0.1593
Caucasian	55	101	
Black	0 (0)	5 (4)	
Hispanic	1 (2)	7 (6)	
North African	0 (0)	1 (1)	
Unknown	0 (0)	4 (3)	
Naive	5 (9)	21 (19)	0.1180
ART regimen ^1^, N (%)			0.6799
NNRTI-based	26	48	
PI-based	12	25	
INSTI-based	4	3	
Other formulations	5 (8)	7 (7)	
Unknown	1 (2)	3 (3)	
Time since HIV diagnosis, years (IQR)	13 (4–19)	7 (2–13)	0.0078
HIV transmission category ^2^, N (%)			0.113
MSW/IDU	16	30	
MSM/IDU	1	1	
MSM	14	21	
MSM/MSW and WSM/WSW	10	41	
Unknown	16 (25)	22 (18)	
CD4 T cell nadir			
CD4 T cell nadir, cells/µL, median (IQR)	264 (159–464)	284 (154–441)	0.851
CD4 T cell nadir < 350 cells/µL, N (%)	35 (65)	21 (62)	0.8222
CD4 T cells counts at the visit, cells/µL, median (IQR)	555 (330–787)	536 (343–717)	0.7366
CD8 T cells counts at the visit, cells/µL, median (IQR)	980 (668–1282]	910 (698–1359)	0.9112
Ratio CD4/CD8	0.59 (0.36–0.79)	0.54 (0.34–0.78)	0.5962
VL, copies/mL, median (IQR)	0 (0–157)	0 (0–18,000)	0.2461
VL < 50 copies/mL, N (%)	38 (68)	71 (62)	0.5014
History of AIDS, N (%)	22 (40)	39 (35)	0.6099
HCV ^3^ Co-infection, N (%)	27 (49)	39 (35)	0.0927
Alcohol consumption, yes, N (%)	11 (21)	26 (24)	0.6956
Smoking, yes, N (%)	34 (62)	60 (54)	0.3253
Obesity and Body Measurements			
BMI ^4^, kg/m^2^, median (IQR)	24.3 (22.6–27.7)	24.3 (22.0–26.4)	0.4880
Obesity (BMI > 30), N (%)	7 (13)	3 (3)	0.0143
Men			
Waist circumference, cm, median (IQR)	89 (83–93)	88 (82–93)	0.6365
Abdominal obesity, N (%)	4 (11)	4 (5)	0.2540
Women			
Waist circumference, cm, median (IQR)	86 (76–94)	80.5 (72.5–83)	0.1752
Abdominal obesity, N (%)	7 (41)	5 (19)	0.1679
Cardiovascular risk factors and lipid profile			
cIMT ^5^, median (IQR)	2.0 (1.6–2.5)	1.0 (0.9–1.1)	<0.0001
COMVIH-COR Score ^6^, median (IQR)	3.5 (0.08–6.91)	2.3 (0.77–3.79)	0.0526
Total cholesterol, mg/dL, median (IQR)	198 (167–222)	172 (152–202)	0.0027
High-Density Lipoprotein, mg/dL, median (IQR)	44 (34.8–57.9)	44.6 (37.9–56.9)	0.5025
Triglycerides, mg/dL, median (IQR)	146 (101–202)	106 (82–157)	0.0042
LDL cholesterol, mg/dL, median (IQR)	116 (93–146)	97 (83–128)	0.0119
Dyslipidemia, N (%)	18 (33)	29 (26)	0.3615
Hypertension, N (%)	54 (47)	37 (33)	0.0414
Diabetes type 2, N (%)	3 (6)	2 (2)	0.3324

Data are presented as median [IQR] or number (percentage). Group comparisons were performed using Mann–Whitney U test for continuous variables and Chi-squared or Fisher’s exact test for categorical variables, as appropriate. ^1^ NNRTI, Non-Nucleoside Reverse Transcriptase Inhibitors, including Efavirenz, Nevirapine, Rilpivirine, Etravirine; PI, Protease Inhibitors including Darunavir, Atazanavir, Lopinavir, Fosamprenavir; INSTI, Integrase Strand Transfer Inhibitors including Dolutegravir, Raltegravir, Elvitegravir; Dual therapy (Dolutegravir + Lamivudine), Other formulations (Zidovudine, Didanosina, Stavudine); ^2^ MSW = Men that have sex with women, MSM = Men that have sex with men, WSM = Women that have sex with men, WSW = Women that have sex with women, IDU = Injecting Drug User. ^3^ HCV = Hepatitis C Virus. ^4^ BMI = Body Mass Index (weight (kg)/height (m)^2^). ^5^ cIMT: carotid Intima–Media Thickness. ^6^ COMVIH-COR Score: estimation of the 10-year risk of a coronary event in Spain, adapted for PWH.

**Table 2:** Clinical, demographic, and cardiovascular characteristics of participants from CoRIS cohort, stratified by cardiovascular risk associated factors (CVRA): CVRA ≥ 3 vs CVRA = 0.

	CVRA ^1^ ≥ 3	CVRA ^1^ = 0	*p*-Values
	N = 19	N = 94	
Age, years, median (IQR)	48 (42–50)	44 (40–52)	0.2802
Sex, Male, N (%)	18 (95)	89 (95)	>0.9999
Ethnicity, N (%)			<0.0001
Caucasian	16 (84)	67 (71)	
North African	1 (5)	5 (5)	
Hispanic	2 (11)	22 (23)	
Unknown	0 (0)	6 (6)	
ART-Naive, N (%)	0 (0)	0 (0)	>0.9999
ART regimen ^2^, N (%)			0.044
NNRTI-based	7 (37)	25 (27)	
PI-based	4 (21)	4 (4)	
INSTI-based	3 (16)	29 (31)	
Other formulations	3 (16)	31 (33)	
Unknown	2 (11)	7 (7)	
Time since HIV diagnosis, years (IQR)	8.1 (3.6–10.6)	6.9 (2.9–10.7)	0.9651
HIV transmission category ^3^, N (%)			
IDU	2 (11)	0 (0)	0.0497
MSM/Bisexual	13 (68)	79 (84)	
MSM/MSW and WSM/WSW	3 (16)	14 (15)	
Unknown	1 (5)	7 (7)	
CD4 T cell nadir			
CD4 T cell nadir, cells/µL, median (IQR)	310 (171–418)	454 (288–676)	0.0117
CD4 T cell nadir < 350 cells/µL, N (%)	11 (58)	31 (33)	0.0147
CD4 T cells counts at the visit, cells/µL, median (IQR)	548 (421–807)	692 (484–921)	0.284
VL, copies/mL, median (IQR)	0 (0–105)	0 (0–0)	0.0826
VL < 50 copies/mL, N (%)	14 (74)	81 (86)	0.1621
History of AIDS, N (%)	3 (16)	6 (6)	0.1744
HCV ^4^ Co-infection, N (%)	2 (11)	0 (0)	0.027
Smoking, yes, N (%)	15	0 (0)	0.0006
Obesity and Body Measurements			
BMI ^5^, kg/m^2^, median (IQR)	33 (30–39)	25 (23–27)	<0.0001
Obesity (BMI > 30), N (%)	16 (84)	0 (0)	<0.0001
Cardiovascular risk factors and lipid profile			
COMVIH-COR Score ^6^	12.9 (7.3–17.3)	2.1 (1.3–3.3)	<0.0001
Total cholesterol, mg/dL, median (IQR)	188 (161–252)	175 (156–199)	0.062
High-Density Lipoprotein, mg/dL, median (IQR)	37 (34–47)	45 (40–53)	0.0058
Triglycerides, mg/dL, median (IQR)	167 (109–266)	101 (140–77)	0.0002
LDL cholesterol, mg/dL, median (IQR)	107 (83–169)	106 (83–124)	0.2323
Dyslipidemia, N (%)	8 (42)	0 (0)	<0.0001
Hypertension, N (%)	15 (79)	0 (0)	<0.0001
Diabetes type 2, N (%)	12 (63)	0 (0)	<0.0001

Data are presented as median [IQR] or number (percentage). Group comparisons were performed using Mann–Whitney U test for continuous variables and Chi-squared or Fisher’s exact test for categorical variables, as appropriate. ^1^ CVRA = cardiovascular risk associated comorbidities (presence of smoking, hypertension, diabetes, obesity, or dyslipidemia). ^2^ NNRTI= Efavirenz, Nevirapine, Rilpivirine, Etravirine, PI = Darunavir, Atazanavir, Lopinavir, Fosamprenavir, INSTI = Dolutegravir, Raltegravir, Elvitegravir, Dual therapy (Dolutegravir + Lamivudina), Other formulations (Zidovudina, Didanosina, Stavudine). ^3^ MSW = Men that have sex with women, MSM = Men that have sex with men, WSM= Women that have sex with men, WSW= Women that have sex with women, IDU= Injecting Drug User. ^4^ HCV = Hepatitis C Virus. ^5^ BMI = Body Mass Index (weight (kg)/height (m)^2^). ^6^ COMVIH-COR Score: estimation of the 10-year risk of a coronary event in Spain, adapted for PWH.

## Data Availability

The data supporting the findings of this study contain sensitive clinical and molecular information from human participants and are therefore not publicly available due to ethical and privacy restrictions. De-identified data may be made available from the corresponding author upon reasonable request and subject to approval by the relevant ethics committees and data governance bodies of the HUMT and CoRIS cohorts.

## Supplementary Materials

The following supporting information can be downloaded at: https://www.mdpi.com/article/10.3390/medsci14010085/s1, Table S1: Clinical, demographic and cardiovascular characteristics of participants from HUMT cohort selected to perform de miArray (N = 72); Table S2: List of miRNAs candidates (N = 44) meeting both statistical significance (unadjusted *p*-value < 0.05) and effect size criteria (|log_2_FC| > 1.2); Table S3: Spearman correlation analyses between circulating miRNAs and carotid intima–media thickness (cIMT) in HUMT participants; Table S4: Clinical, demographic and cardiovascular characteristics of participants from HUMT cohort participants that meet COMVIH-CoR criteria (≥10% risk or ≤5% risk); Table S5: Spearman correlation analyses between circulating miRNAs and cardiometabolic variables in the HUMT and CoRIS cohorts; Table S6: Multivariable linear regression models assessing the association between traditional cardiovascular risk factors and circulating miRNA levels in the HUMT and CoRIS cohorts; Table S7: Correlation miRNA expression with HIV-related parameters; Figure S1: Comparison of endogenous miRNA control expression between cohorts; Figure S2: Heatmaps comparing miRNAs levels among comorbidities; Figure S3: Venn-Diagram regarding the atherosclerosis status and COMVIH-CoR punctuation among HUMT individuals.

## Abbreviations

The following abbreviations are used in this manuscript:

PWH	PWH
miRNA	Micro-RNA
CVD	Cardiovascular disease
AP	Atheroma plaque
CVRA	cardiovascular risk associated factors
ART	Antiretroviral therapy
cIMT	Carotid intima–media thickness
PBMCs	peripheral blood mononuclear cells
RT-qPCR	performed reverse transcription quantitative PCR
NNRTI	Non-Nucleoside Reverse Transcriptase Inhibitors
PI	Protease Inhibitors
INSTI	Integrase Strand Transfer Inhibitors
MSW	Men that have sex with women
MSM	Men that have sex with men
WSM	Women that have sex with men
WSW	Women that have sex with women
IDU	Injecting Drug User
HCV	Hepatitis C Virus.
BMI	Body Mass Index
PGK1	phosphoglycerate kinase 1
TLR	toll-like receptors
PPARγ	proliferator–activated receptor gamma
ATP2B1	calcium-transporting ATPase 1
