# Supplementary material for: Circulating miRNA Signatures Associated with Atherosclerosis and Cardiometabolic Comorbidities in People with HIV
Source: Med Sci (Basel). 2026 Feb 12;14(1):85. doi: 10.3390/medsci14010085 (PMC12922024; doi:10.3390/medsci14010085)
Supplement: Supplementary file 1 [file medsci-14-00085-s001.zip › medsci-4119591-supplementary.pdf]

# Supplementary Material:

# Circulating miRNA Signatures Associated With Atherosclerosis and Cardiometabolic Comorbidities in People With HIV

Marina Martinez-Velasco, Jose Francisco Sanchez-Herrero, Laura Ibañez, Pablo Velli, Francisco Manuel Muñoz-Lopez, Mireia Cairó, Angeles Jaen, Roser Font, Xavier Martinez-Lacasa, Josep Royo, Joaquim Peraire, Naya Faro-Míguez, Antonio Rivero, Julián Olalla, Pilar Ruiz-Seco, Luis Fernando López-Cortés, Lauro Sumoy, Marta Massanella David Dalmau on behalf of the HUMT and CoRIS cohorts.

**Table S1. Clinical, demographic and cardiovascular characteristics of participants from HUMT cohort selected to perform de miArray (N=72).**

| | AP+ N=36 | AP- N=36 | P-values |
|---|---|---|---|
| Age, years, median (IQR) | 48.5 (46-51) | 41.5 (37-47) | <0.0001 |
| Sex, Male, N (%) | 30 (83) | 31 (86) | >0.9999 |
| Ethnicity, N (%) | | | |
| Caucasian | 36 (100) | 28 (78) | 0.0051 |
| Black | 0 (0) | 3 (8) | |
| Hispanic | 0 (0) | 1 (3) | |
| Unknown | 0 (0) | 4 (11) | |
| ART-Naive, N (%) | 1 (3) | 4 (11) | 0.357 |
| ART regimen[1], N (%) | | | |
| NNRTI-based | 19 (53) | 22 (61) | |
| PI-based | 11 (30) | 9 (35) | |
| INSTI-based | 3 (8) | 0 (0) | |
| Other formulations | 2 (6) | 1 (3) | |
| Unknown | 1 (3) | 4 (11) | |
| Time since HIV diagnosis, years (IQR) | 13 (7-17) | 8 (2-14.5) | 0.0279 |
| HIV transmission category[2], N (%) | | | 0.2542 |
| MSW/IDU | 8 (22) | 12 (33) | |
| MSM/IDU | 1 (3) | 1 (3) | |
| MSM | 10 (28) | 3 (8) | |
| MSM/MSW and WSM/WSW | 10 (28) | 13 (36) | |
| Unknown | 7 (19) | 7 (19) | |
| CD4 T cell nadir | | | |
| CD4 T cell nadir, cells/µL, median (IQR) | 212 (122-364) | 324 (146-448) | 0.1732 |
| CD4 T cell nadir <350 cells/µl, N (%) | 26 (55) | 21 (45) | 0.3088 |
| CD4 T cells counts at the visit, cells/µl, median (IQR) | 573 (358-809) | 517 (371-748) | 0.7391 |
| CD8 T cells counts at the visit, cells/µl, median (IQR) | 958 (723-1373) | 1001 (656-1358) | 0.6522 |
| Ratio CD4/CD8 | 0.59-0.38-0.79) | 0.52 (0.32-0.83) | 0.9065 |
| VL, copies/mL, median (IQR) | 0 (0-0) | 0 (0-13577) | 0.0723 |
| VL <50 copies/mL, N (%) | 29 (80) | 23 (64) | 0.1877 |
| History of AIDS, N (%) | 18 (50) | 12 (33) | 0.2318 |
| HCV[3] Co-infection, N (%) | 15 (42) | 12 (33) | 0.6268 |
| Alcohol consumption, yes, N(%) | 8 (22) | 11 (31) | 0.5936 |
| Smoking, yes, N (%) | 21 (58) | 22 (61) | >0.9999 |
| Obesity and Body Measurements | | | |
| BMI[4], kg/m², median (IQR) | 23.66 (22.73-26.92) | 23.59 (21.26-26.27) | 0.36 |
| Obesity (BMI>30), N (%) | 5 (17) | 0 (0) | 0.0217 |
| *Men* | | | |
| Waist circumference, cm, median (IQR) | 88.5 (82.0-93.25) | 86.0 (81.5-92.5) | 0.4086 |
| Abdominal obesity, N (%) | 4 (13) | 0 (0) | 0.1124 |
| *Women* | | | |
| Waist circumference, cm, median (IQR) | 84 (72.25-92.5) | 70 (66.5-76.5) | 0.0866 |
| Abdominal obesity, N (%) | 1 (17) | 0 (0) | >0.9999 |
| Cardiovascular risk factors and lipid profile | | | |
| cIMT[5] | 2.25 (2.0-2.87) | 0.8 (0.8-0.9) | <0.0001 |
| COMVIH-COR Score[6] | 3.69 (1.15-5.98) | 1.9 (1.1-3.33) | 0.0344 |
| Total cholesterol, mg/dL, median (IQR) | 196 (163-231) | 172 (149-201) | 0.0238 |
| High-Density Lipoprotein, mg/dL, median (IQR) | 57 (34-56) | 44 (39-63) | 0.6684 |
| Triglycerides, mg/dL, median (IQR) | 142 (114-180) | 109 (91-159) | 0.0527 |
| LDL cholesterol, mg/dL, median (IQR) | 116 (86-158) | 95 (78-129) | 0.0655 |
| Dyslipidemia, N (%) | 13 (37) | 5 (14) | 0.0539 |
| Hypertension, N (%) | 19 (53) | 10 (28) | 0.0537 |
| Diabetes type 2, N (%) | 2 (6) | 1 (3) | >0.9999 |

Data is presented as median (IQR) or number (percentage). Group comparisons were performed using Mann–Whitney U test for continuous variables and Chi-squared or Fisher's exact test for categorical variables, as appropriate. [1] NNRTI= Efavirenz, Nevirapine, Rilpivirine, Etravirine, PI=Darunavir, Atazanavir, Lopinavir, Fosamprenavir, INSTI=Dolutegravir, Raltegravir, Elvitegravir, Dual therapy (Dolutegravir + Lamivudine), Other formulations (Zidovudine, Didanosine, Stavudine); [2]MSW=Men that have sex with women, MSM=Men that have sex with men, WSM= Women that have sex with men, WSW= Women that have sex with women, IDU= Injecting Drug User. [3]HCV=Hepatitis C Virus. [4]BMI=Body Mass Index (weight (kg)/height (m)2) [5]cIMT: carotid Intima-Media Thickness [6]COMVIH-COR Score: estimation of the 10-year risk of a coronary event, adapted for PWH.

**<u>Table S2:</u> List of miRNAs candidates (N=44) meeting both statistical significance (unadjusted p-value<0.05) and effect size criteria (|$log_2$FC| > 1.2).**

| miRNA | log(FoldChange) | Average Expression | *p-values* |
|---|---|---|---|
| hsa-miR-223-5p | 0.275183337 | 0.290339034 | 0.001177017 |
| hsa-miR-652-5p | 1.040869011 | 2.853663084 | 0.003590355 |
| **hsa-miR-3613-5p[$]** | **2.197427179** | **6.850295926** | **0.003662317** |
| hsa-miR-1180-5p | -0.29483273 | 1.161616402 | 0.004662321 |
| hsa-miR-501-5p | 0.675196282 | 1.773854818 | 0.006827169 |
| hsa-miR-505-3p | 0.416293778 | 0.649528992 | 0.007709109 |
| hsa-miR-324-5p | 0.63347014 | 1.593066544 | 0.007757242 |
| hsa-miR-4743-5p | 0.555102268 | 2.080787166 | 0.008269866 |
| hsa-miR-4433-5p | -0.332009463 | 1.100357024 | 0.01004667 |
| hsa-miR-1299 | 0.295819148 | 0.413553981 | 0.012825357 |
| hsa-miR-487a-3p | 0.593724191 | 0.74226163 | 0.012941446 |
| hsa-miR-326 | 0.619423045 | 2.015184073 | 0.013104439 |
| hsa-miR-4307 | 1.094291756 | 1.407407993 | 0.01614483 |
| hsa-miR-1287-5p | 0.429103627 | 0.920772138 | 0.016804946 |
| **hsa-miR-4668-3p[$]** | **1.719445067** | **3.086314763** | **0.018064722** |
| hsa-miR-150-3p | -0.588374666 | 4.830988804 | 0.018123423 |
| hsa-miR-6808-3p | -0.483514111 | 4.196246948 | 0.022937392 |
| hsa-miR-6516-5p | 0.297140118 | 0.886973527 | 0.02347866 |
| **hsa-miR-638[$]** | **-0.411474764** | **8.257746845** | **0.026267825** |
| hsa-miR-3154 | 0.2862061 | 0.545350843 | 0.027160006 |
| hsa-miR-4763-3p | -0.457833796 | 5.690622371 | 0.028702815 |
| hsa-miR-548a-3p | 0.364519922 | 0.980519662 | 0.03060296 |
| hsa-miR-6807-5p | 0.704242878 | 2.608980674 | 0.031520753 |
| hsa-miR-301a-3p | 0.387279442 | 0.599269452 | 0.03353481 |
| **hsa-miR-140-5p[$]** | **0.683868637** | **2.596087988** | **0.033958167** |
| hsa-miR-5001-5p | -0.356573146 | 5.904777761 | 0.035064492 |
| hsa-miR-6729-5p | -0.425005191 | 8.9939727 | 0.035676357 |
| hsa-miR-5100 | -0.585824325 | 5.700033023 | 0.036088135 |
| hsa-miR-4775 | 1.164633209 | 1.96989558 | 0.036491556 |
| hsa-miR-1275 | -0.450566956 | 5.43684621 | 0.036632559 |
| hsa-miR-628-5p | 0.285506906 | 1.063952881 | 0.036834483 |
| hsa-miR-6787-5p | -0.425592551 | 4.459216309 | 0.039255719 |
| hsa-miR-378e | 0.411777387 | 1.118888597 | 0.041404097 |
| hsa-miR-7704 | -0.350569114 | 9.84838538 | 0.042651367 |
| hsa-miR-150-5p | -0.488784652 | 11.18638253 | 0.045167855 |
| hsa-miR-3667-5p | 0.32290022 | 1.331049468 | 0.045180296 |
| hsa-miR-338-5p | 0.369662884 | 1.35933844 | 0.04602386 |
| hsa-miR-6126 | -0.470030425 | 5.771988792 | 0.047453607 |
| hsa-miR-186-3p | 0.712645658 | 1.559327913 | 0.047569773 |
| hsa-miR-4758-5p | -0.399675567 | 4.832703093 | 0.048362056 |
| hsa-miR-543 | 0.344666127 | 0.772024529 | 0.048536242 |
| hsa-miR-6869-5p | -0.341349977 | 9.795455703 | 0.048978754 |
| hsa-miR-7114-5p | 0.556135333 | 2.210520603 | 0.048988619 |
| hsa-miR-4466 | -0.328381614 | 8.905435192 | 0.04956655 |

[$]miRNAs selected for validation

**Table S3. Spearman correlation analyses between circulating miRNAs and carotid intima–media thickness (cIMT) in HUMT participants**

| Analysis | miRNA | Spearman r | p-values | N |
|---|---|---|---|---|
| cIMT (mm) *All participants* | miR-140-5p | -0.0099 | 0.89 | 170 |
| | miR-146b-5p | 0.0369 | 0.63 | 170 |
| | miR-27b-5p | 0.0331 | 0.67 | 164 |
| | miR-3613-5p | 0.0220 | 0.78 | 169 |
| | miR-638 | -0.0378 | 0.63 | 169 |
| cIMT (mm) *Only men* | miR-140-5p | 0.0306 | 0.73 | 127 |
| | miR-146b-5p | 0.0960 | 0.28 | 127 |
| | miR-27b-5p | 0.0843 | 0.36 | 121 |
| | miR-3613-5p | 0.1138 | 0.20 | 126 |
| | miR-638 | -0.0152 | 0.87 | 126 |
| cIMT (mm) *Only women* | miR-140-5p | -0.0982 | 0.53 | 43 |
| | miR-146b-5p | -0.1752 | 0.26 | 43 |
| | miR-27b-5p | -0.1206 | 0.44 | 43 |
| | miR-3613-5p | -0.2862 | 0.06 | 43 |
| | miR-638 | -0.1517 | 0.33 | 43 |

**Table S4.** **Clinical, demographic and cardiovascular characteristics of participants from HUMT cohort participants that meet COMVIH-CoR criteria (≥10% risk or ≤5% risk).**

| | COMVIH-CoR score ≥ 10% N=14 | COMVIH-CoR score ≤ 5% N=125 | p-values |
|---|---|---|---|
| Age, years, median (IQR) | 53.5 (47.7-60.5) | 42 (38-47.5) | <0.0001 |
| Sex, Male, N (%) | 12 (86) | 95 (76) | 0.5220 |
| Ethnicity, N (%) | | | |
| Caucasian | 13 (93) | 113 (90) | 0.7648 |
| Black | 0 (0) | 4 (3) | |
| Hispanic | 1 (7) | 7 (6) | |
| North African | 0 (0) | 1 (1) | |
| Naive | 0 (0) | 22 (18) | 0.0666 |
| ART regimen[1], N (%) | | | 0.0174 |
| NNRTI-based | 4 (29) | 58 (46) | |
| PI-based | 6 (43) | 28 (22) | |
| INSTI-based | 3 (21) | 4 (3) | |
| Other formulations | 0 (0) | 11 (9) | |
| Unknown | 1 (7) | 2 (2) | |
| Time since HIV diagnosis, years (IQR) | 12 (8.5-18) | 7 (2.75-15) | 0.0241 |
| HIV transmission category[2], N (%) | | | |
| MSW/IDU | 5 (36) | 35 (28) | 0.4241 |
| MSM/IDU | 1 (7) | 1 (1) | |
| MSM | 2 (14) | 27 (22) | |
| MSM/MSW and WSM/WSW | 6 (43) | 36 (29) | |
| Unknown | 5 (36) | 26 (21) | |
| CD4 T cell nadir | | | |
| CD4 T cell nadir, cells/μL, median (IQR) | 245 (135-405) | 284 (169-476) | 0.3662 |
| CD4 T cell nadir <350 cells/μl, N (%) | 9 (64) | 71 (57) | 0.7796 |
| CD4 T cells counts at the visit, cells/μl, median (IQR) | 412 (288-959) | 546 (330-733) | 0.9962 |
| CD8 T cells counts at the visit, cells/μl, median (IQR) | 1053 (576-1554) | 928 (668-1313) | 0.9352 |
| Ratio CD4/CD8 | 0.66 (0.2-1.2) | 0.54 (0.34-0.78) | 0.4902 |
| VL, copies/mL, median (IQR) | 0 (0-0) | 0 (0-7970) | 0.0090 |
| VL <50 copies/mL, N (%) | 13 (93) | 75 (60) | 0.0176 |
| History of AIDS, N (%) | 6 (43) | 43 (34) | 0.3043 |
| HCV[3] Co-infection, N (%) | 2 (14) | 50 (40) | 0.0716 |
| Alcohol consumption, yes, N(%) | 3 (21) | 27 (22) | 0.6379 |
| Smoking, yes, N (%) | 8 (57) | 72 (55) | 0.043 |
| Obesity and Body Measurements | | | |
| BMI[4], kg/m², median (IQR) | 23.6 (22.7-26.9) | 23.6 (21.2-26.2) | 0.3617 |
| Obesity (BMI>30), N (%) | 2 (14) | 7 (6) | 0.1466 |
| *Men* | | | |
| Waist circumference, cm, median (IQR) | 90 (84-95) | 87 (82-93) | 0.3398 |
| Abdominal obesity, N (%) | 1 (7) | 4 (3) | 0.3111 |
| *Women* | | | |
| Waist circumference, cm, median (IQR) | 99 (86-112) | 81 (72-88) | 0.1109 |
| Abdominal obesity, N (%) | 1 (7) | 9 (7) | 0.5343 |
| Cardiovascular risk factors and lipid profile | | | |
| Atheroma plaque, yes, N(%) | 11 (79) | 31 (25) | <0.0001 |
| cIMT[5] | 1.95 (1.45-3.1) | 1.1 (0.9-1.35) | <0.0001 |
| Total cholesterol, mg/dL, median (IQR) | 215 (188-231) | 171 (149-199) | <0.0001 |
| High-Density Lipoprotein, mg/dL, median (IQR) | 33 (28-42) | 48 (38-59) | 0.0005 |
| Triglycerides, mg/dL, median (IQR) | 261 (156-360) | 104 (81-150) | <0.0001 |
| LDL cholesterol, mg/dL, median (IQR) | 139 (116-177) | 97 (79-120) | <0.0001 |
| Dyslipidemia, N (%) | 5 (36) | 32 (26) | 0.7263 |
| Hypertension, N (%) | 13 (93) | 28 (22) | <0.0001 |
| Diabetes type 2, N (%) | 2(14) | 3 (2) | 0.0497 |

Data is presented as median (IQR) or number (percentage). Group comparisons were performed using Mann–Whitney U test for continuous variables and Chi-squared or Fisher's exact test for categorical variables, as appropriate. [1] NNRTi= Efavirenz, Nevirapine, Rilpivirine, Etravirine, PI=Darunavir, Atazanavir, Lopinavir, Fosamprenavir, INSTI=Dolutegravir, Raltegravir, Elvitegravir, Dual therapy (Dolutegravir + Lamivudina), Other formulation (Zidovudine, Didanosine, Stavudine); [2]MSW=Men that have sex with women, MSM=Men that have sex with men, WSM= Women that have sex with men, WSW= Women that have sex with women, IDU= Injecting Drug User. [3]HCV=Hepatitis C Virus. [4]BMI=Body Mass Index (weight (kg)/height (m)²). [5]cIMT: carotid Intima-Media Thickness

**Table S5.** **Spearman correlation analyses between circulating miRNAs and cardiometabolic variables in the HUMT and CoRIS cohorts.**

| Analysis | Cohort | miRNA | Spearman r | p-values | N |
|---|---|---|---|---|---|
| Age (years) | HUMT | miR-140-5p | 0.0019 | 0.98 | 170 |
| | | miR-146b-5p | 0.0331 | 0.67 | 170 |
| | | miR-27b-5p | -0.0220 | 0.78 | 164 |
| | | miR-3613-5p | 0.0088 | 0.91 | 169 |
| | | miR-638 | -0.1635 | 0.03 | 169 |
| Total cholesterol (mg/dL) | HUMT | miR-140-5p | 0.1505 | 0.05 | 170 |
| | | miR-146b-5p | 0.1512 | 0.05 | 170 |
| | | miR-27b-5p | 0.0363 | 0.64 | 164 |
| | | miR-3613-5p | 0.0563 | 0.47 | 169 |
| | | miR-638 | 0.0020 | 0.98 | 169 |
| LDL cholesterol (mg/dL) | HUMT | miR-140-5p | 0.1421 | 0.06 | 170 |
| | | miR-146b-5p | 0.0919 | 0.23 | 170 |
| | | miR-27b-5p | 0.0461 | 0.56 | 164 |
| | | miR-3613-5p | 0.0258 | 0.74 | 169 |
| | | miR-638 | 0.0661 | 0.39 | 169 |
| High-Density Lipoprotein (mg/dL) | HUMT | miR-140-5p | 0.0944 | 0.22 | 170 |
| | | miR-146b-5p | 0.1472 | 0.06 | 170 |
| | | miR-27b-5p | 0.1808 | 0.02 | 164 |
| | | miR-3613-5p | 0.0460 | 0.55 | 169 |
| | | miR-638 | 0.0264 | 0.73 | 169 |
| Triglycerids (mg/dL) | HUMT | miR-140-5p | 0.0105 | 0.89 | 170 |
| | | miR-146b-5p | -0.0207 | 0.79 | 170 |
| | | miR-27b-5p | -0.1080 | 0.17 | 164 |
| | | miR-3613-5p | -0.0101 | 0.90 | 169 |
| | | miR-638 | -0.0654 | 0.40 | 169 |
| COMVIH-CoR Score | HUMT | miR-140-5p | 0.0891 | 0.25 | 170 |
| | | miR-146b-5p | -0.1247 | 0.11 | 170 |
| | | miR-27b-5p | -0.0158 | 0.84 | 164 |
| | | miR-3613-5p | -0.0365 | 0.64 | 169 |
| | | miR-638 | -0.1396 | 0.07 | 169 |
| Age (years) | CoRIS | miR-140-5p | 0.1142 | 0.23 | 112 |
| | | miR-146b-5p | -0.0504 | 0.60 | 113 |
| | | miR-27b-5p | 0.1260 | 0.19 | 111 |
| | | miR-3613-5p | -0.0138 | 0.88 | 113 |
| | | miR-638 | -0.0110 | 0.91 | 113 |
| Total cholesterol (mg/dL) | CoRIS | miR-140-5p | -0.0606 | 0.53 | 110 |
| | | miR-146b-5p | -0.0137 | 0.89 | 111 |
| | | miR-27b-5p | -0.0037 | 0.97 | 109 |
| | | miR-3613-5p | -0.0341 | 0.72 | 111 |
| | | miR-638 | -0.0075 | 0.94 | 111 |
| LDL cholesterol (mg/dL) | CoRIS | miR-140-5p | -0.0690 | 0.47 | 110 |
| | | miR-146b-5p | -0.0827 | 0.39 | 111 |
| | | miR-27b-5p | -0.0245 | 0.80 | 109 |
| | | miR-3613-5p | -0.0148 | 0.88 | 111 |
| | | miR-638 | -0.0360 | 0.71 | 111 |
| High-Density Lipoprotein (mg/dL) | CoRIS | miR-140-5p | -0.1314 | 0.17 | 110 |
| | | miR-146b-5p | 0.1066 | 0.27 | 111 |
| | | miR-27b-5p | 0.0233 | 0.81 | 109 |
| | | miR-3613-5p | -0.0940 | 0.33 | 111 |
| | | miR-638 | -0.0191 | 0.84 | 111 |
| Triglycerids (mg/dL) | CoRIS | miR-140-5p | 0.0359 | 0.71 | 110 |
| | | miR-146b-5p | -0.0808 | 0.40 | 111 |
| | | miR-27b-5p | 0.0016 | 0.99 | 109 |
| | | miR-3613-5p | -0.0244 | 0.80 | 111 |
| | | miR-638 | 0.0823 | 0.39 | 111 |
| COMVIH-CoR Score | CoRIS | miR-140-5p | 0.2121 | 0.02 | 112 |
| | | miR-146b-5p | 0.1260 | 0.18 | 113 |
| | | miR-27b-5p | 0.1425 | 0.14 | 111 |
| | | miR-3613-5p | 0.0983 | 0.30 | 113 |
| | | miR-638 | 0.1037 | 0.27 | 113 |

**Table S6. Multivariable linear regression models assessing the association between traditional cardiovascular risk factors and circulating miRNA levels in the HUMT and CoRIS cohorts.**

| miRNA | Cohort | Covariate | β (Estimate) | Std. Error | t-value | p-value | Adjusted $R^2$ | Model p-value |
|---|---|---|---|---|---|---|---|---|
| **miR-140-5p** | HUMT | Intercept | 0.84 | 0.289 | 2.91 | 0.004 | 0.01 | 0.299 |
| | | Smokers | -0.187 | 0.109 | -1.714 | 0.089 | | |
| | | Obese | -0.223 | 0.22 | -1.014 | 0.312 | | |
| | | Hypertensive | 0.176 | 0.11 | 1.6 | 0.112 | | |
| | | Diabetics | -0.11 | 0.33 | -0.333 | 0.74 | | |
| | | Dyslipidemic | -0.098 | 0.119 | -0.823 | 0.412 | | |
| | | Cholesterol | 0.001 | 0.001 | 0.7 | 0.485 | | |
| | | HDL | -0.001 | 0.003 | -0.399 | 0.691 | | |
| **miR-146b-5p** | HUMT | Intercept | 0.667 | 0.181 | 3.682 | <0.001 | 0.008 | 0.322 |
| | | Smokers | -0.005 | 0.068 | -0.077 | 0.939 | | |
| | | Obese | -0.172 | 0.138 | -1.247 | 0.214 | | |
| | | Hypertensive | 0.033 | 0.069 | 0.477 | 0.634 | | |
| | | Diabetics | -0.233 | 0.207 | -1.126 | 0.262 | | |
| | | Dyslipidemic | -0.05 | 0.075 | -0.674 | 0.501 | | |
| | | Cholesterol | 0.001 | 0.001 | 0.772 | 0.442 | | |
| | | HDL | 0.003 | 0.002 | 1.482 | 0.14 | | |
| **miR-27b-5p** | HUMT | Intercept | -7.128 | 0.237 | -30.05 | <0.001 | 0.09 | 0.004 |
| | | **Smokers** | **-0.303** | **0.09** | **-3.373** | **0.001** | | |
| | | Obese | -0.156 | 0.18 | -0.864 | 0.389 | | |
| | | **Hypertensive** | **0.205** | **0.091** | **2.266** | **0.025** | | |
| | | Diabetics | 0.146 | 0.271 | 0.536 | 0.593 | | |
| | | Dyslipidemic | 0.016 | 0.098 | 0.162 | 0.872 | | |
| | | Cholesterol | -0.0004 | 0.001 | -0.371 | 0.711 | | |
| | | HDL | 0.005 | 0.003 | 1.768 | 0.079 | | |
| **miR-3613-5p** | HUMT | Intercept | -3.935 | 0.205 | -19.188 | <0.001 | -0.021 | 0.798 |
| | | Smokers | -0.091 | 0.078 | -1.171 | 0.244 | | |
| | | Obese | -0.142 | 0.156 | -0.909 | 0.365 | | |
| | | Hypertensive | 0.097 | 0.078 | 1.235 | 0.219 | | |
| | | Diabetics | -0.086 | 0.235 | -0.367 | 0.714 | | |
| | | Dyslipidemic | 0.038 | 0.085 | 0.451 | 0.653 | | |
| | | Cholesterol | -0.0004 | 0.001 | -0.423 | 0.673 | | |
| | | HDL | 0.001 | 0.002 | 0.512 | 0.61 | | |
| **miR-638** | HUMT | Intercept | 0.051 | 0.005 | 9.407 | <0.001 | 0.035 | 0.094 |
| | | Smokers | -0.003 | 0.002 | -1.576 | 0.117 | | |
| | | **Obese** | **-0.009** | **0.004** | **-2.257** | **0.026** | | |
| | | Hypertensive | 0.0003 | 0.002 | 0.14 | 0.889 | | |
| | | **Diabetics** | **-0.012** | **0.006** | **-2.017** | **0.045** | | |
| | | Dyslipidemic | 0.0004 | 0.002 | 0.191 | 0.849 | | |
| | | Cholesterol | -0.0000005 | 0.00003 | -0.018 | 0.985 | | |
| | | HDL | -0.00004 | 0.00006 | -0.693 | 0.489 | | |

| | | | | | | | | |
|---|---|---|---|---|---|---|---|---|
| miR-140-5p | CoRIS | Intercept | 1.50 | 0.28 | 5.33 | <0.0001 | 0.0689 | 0.0448 |
| | | Smokers | −0.23 | 0.30 | −0.79 | 0.429 | | |
| | | Obese | 0.35 | 0.32 | 1.11 | 0.269 | | |
| | | Hypertensive | −0.10 | 0.58 | −0.18 | 0.854 | | |
| | | Diabetics | 0.39 | 0.31 | 1.26 | 0.211 | | |
| | | Dyslipidemic | 0.20 | 0.33 | 0.61 | 0.542 | | |
| | | Cholesterol | −0.002 | 0.00 | −1.18 | 0.242 | | |
| | | HDL | −0.002 | 0.00 | −0.47 | 0.638 | | |
| miR-146b-5p | CoRIS | Intercept | 0.30 | 0.46 | 0.65 | 0.518 | 0.0072 | 0.3597 |
| | | Smokers | −0.31 | 0.50 | −0.61 | 0.543 | | |
| | | Obese | −0.07 | 0.53 | −0.14 | 0.89 | | |
| | | Hypertensive | 0.29 | 0.96 | 0.30 | 0.764 | | |
| | | Diabetics | 0.42 | 0.52 | 0.80 | 0.424 | | |
| | | Dyslipidemic | 0.34 | 0.54 | 0.63 | 0.529 | | |
| | | Cholesterol | −0.013 | 0.00 | −1.38 | 0.172 | | |
| | | HDL | 0.01 | 0.01 | 0.87 | 0.386 | | |
| miR-27b-5p | CoRIS | Intercept | −0.05 | 0.41 | −0.129 | 0.897 | −0.0185 | 0.6559 |
| | | Smokers | 0.17 | 0.43 | 0.40 | 0.694 | | |
| | | Obese | 0.21 | 0.45 | 0.45 | 0.652 | | |
| | | Hypertensive | −0.31 | 0.83 | −0.37 | 0.712 | | |
| | | Diabetics | 0.41 | 0.45 | 0.90 | 0.368 | | |
| | | Dyslipidemic | 0.19 | 0.47 | 0.40 | 0.694 | | |
| | | Cholesterol | −0.0012 | 0.00 | −0.51 | 0.61 | | |
| | | HDL | 0.00 | 0.01 | 0.78 | 0.439 | | |
| miR-3613-5p | CoRIS | Intercept | 0.30 | 0.63 | 0.48 | 0.632 | −0.0103 | 0.557 |
| | | Smokers | −0.30 | 0.68 | −0.45 | 0.653 | | |
| | | Obese | 0.32 | 0.71 | 0.45 | 0.657 | | |
| | | Hypertensive | 0.10 | 1.29 | 0.08 | 0.936 | | |
| | | Diabetics | 0.48 | 0.70 | 0.69 | 0.492 | | |
| | | Dyslipidemic | 0.14 | 0.73 | 0.19 | 0.85 | | |
| | | Cholesterol | −0.0012 | 0.00 | −0.35 | 0.725 | | |
| | | HDL | −0.0036 | 0.01 | −0.40 | 0.692 | | |
| miR-638 | CoRIS | Intercept | 1.05 | 0.22 | 4.76 | <0.0001 | 0.1295 | 0.003 |
| | | **Smokers** | **−0.52** | **0.24** | **−2.18** | **0.0318** | | |
| | | Obese | 0.09 | 0.25 | 0.35 | 0.731 | | |
| | | Hypertensive | 0.42 | 0.46 | 0.91 | 0.364 | | |
| | | Diabetics | 0.27 | 0.25 | 1.11 | 0.272 | | |
| | | Dyslipidemic | 0.08 | 0.26 | 0.31 | 0.756 | | |
| | | Cholesterol | 0.00 | 0.00 | 0.19 | 0.85 | | |
| | | HDL | −0.0019 | 0.00 | −0.60 | 0.551 | | |

Each row shows the regression coefficient (β), standard error, t-value and p-value for the association between the indicated covariate and the transformed expression of the specified miRNA, adjusted for smoking status, obesity, hypertension, diabetes, dyslipidemia, total cholesterol and HDL cholesterol. Statistically significant associations ($p < 0.05$) are highlighted in bold.

**Table S7: Correlation miRNA expression with HIV-related parameters**

| Analysis | Cohort | miRNA | Spearman r | p value | N |
|---|---|---|---|---|---|
| CD4 at inclusion (cel/µL) | HUMT | miR-140-5p | -0.121 | 0.115 | 170 |
| | | miR-146b-5p | 0.188 | 0.0143 | 170 |
| | | miR-27b-5p | -0.048 | 0.545 | 164 |
| | | miR-3613-5p | -0.206 | 0.00708 | 169 |
| | | miR-638 | -0.082 | 0.29 | 169 |
| CD4 nadir (cel/µL) | HUMT | miR-140-5p | -0.095 | 0.225 | 165 |
| | | miR-146b-5p | 0.087 | 0.268 | 165 |
| | | miR-27b-5p | 0.02 | 0.805 | 159 |
| | | miR-3613-5p | -0.092 | 0.243 | 164 |
| | | miR-638 | 0.089 | 0.257 | 164 |
| Years since diagnosis | HUMT | miR-140-5p | -0.091 | 0.262 | 155 |
| | | miR-146b-5p | 0.022 | 0.79 | 155 |
| | | miR-27b-5p | -0.14 | 0.0884 | 150 |
| | | miR-3613-5p | -0.06 | 0.462 | 154 |
| | | miR-638 | -0.112 | 0.165 | 154 |
| CD4 at inclusion (cel/µL) | CoRIS | miR-140-5p | -0.007 | 0.947 | 94 |
| | | miR-146b-5p | 0.232 | 0.0239 | 95 |
| | | miR-27b-5p | -0.092 | 0.381 | 93 |
| | | miR-3613-5p | -0.082 | 0.428 | 95 |
| | | miR-638 | -0.1 | 0.336 | 95 |
| CD4 nadir (cel/µL) | CoRIS | miR-140-5p | -0.103 | 0.285 | 110 |
| | | miR-146b-5p | -0.014 | 0.885 | 111 |
| | | miR-27b-5p | -0.15 | 0.12 | 109 |
| | | miR-3613-5p | -0.174 | 0.0674 | 111 |
| | | miR-638 | -0.152 | 0.11 | 111 |
| Years since diagnosis | CoRIS | miR-140-5p | -0.056 | 0.563 | 110 |
| | | miR-146b-5p | 0.076 | 0.427 | 111 |
| | | miR-27b-5p | -0.087 | 0.366 | 109 |
| | | miR-3613-5p | -0.043 | 0.654 | 111 |
| | | miR-638 | -0.117 | 0.222 | 111 |

**Figure S1**

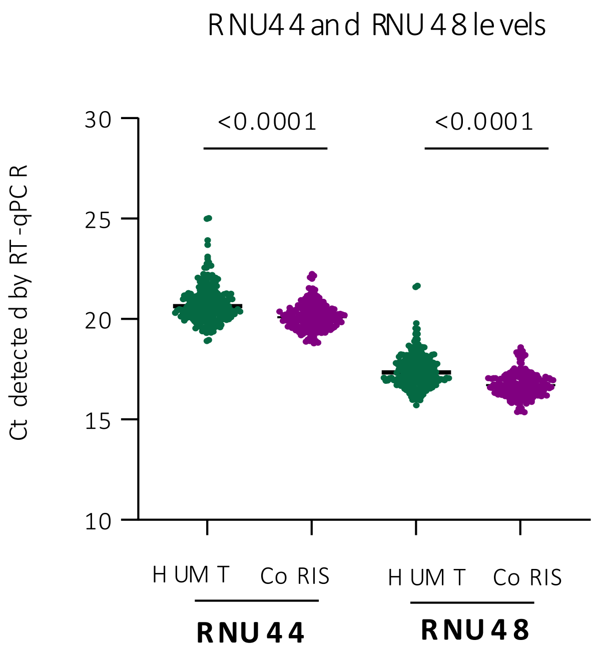


**Figure S1. Comparison of endogenous miRNA control expression between cohorts**. Ct values of RNU44 and RNU48 measured by RT-qPCR differed significantly between the HUMT and CoRIS cohorts (Mann–Whitney U test, p<0.0001 for both endogenous miRNAs).

# Figure S2

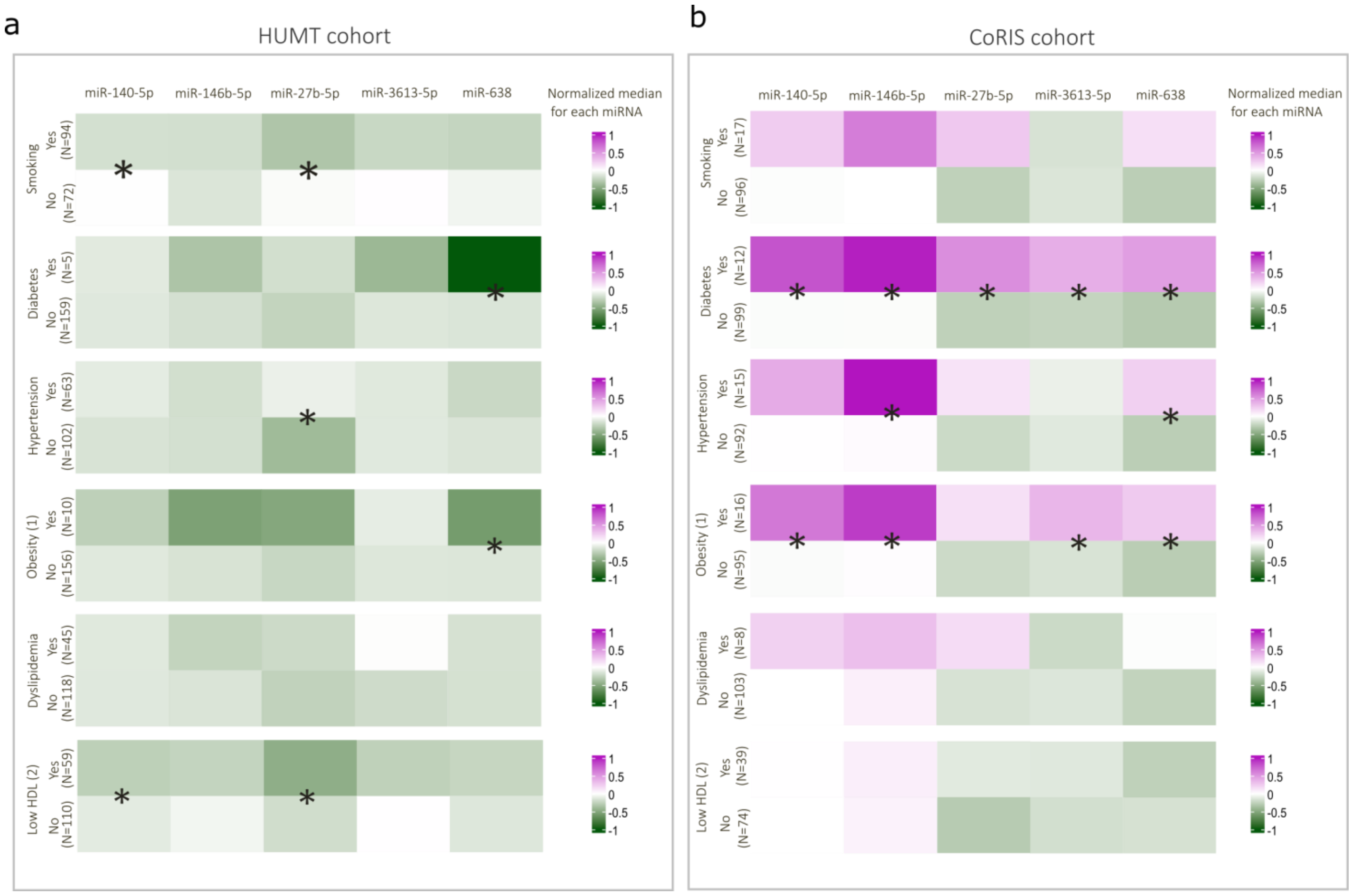


**Figure S2. Heatmap analysis of circulating miRNAs according to cardiovascular associated risk factors in HUMT and CoRIS cohorts.** *(1): Obesity = Body Mass Index ≥30. (2): Low HDL= Individuals with ≤40mg/dL of High-Density Lipoprotein.* **(a)** Heatmap showing expression of miRNAs in HUMT individuals among the different cardiovascular associated risk factors. **(b)** Heatmap showing expression of miRNAs in CoRIS individuals among the different cardiovascular associated risk factors. Values are normalized within the scale of colors and median is represented in the heatmap. Differences between groups analyzed are marked with * (unadjusted $p<0.05$) . Mann-Whitney test was performed to compare groups.

**Figure S3**

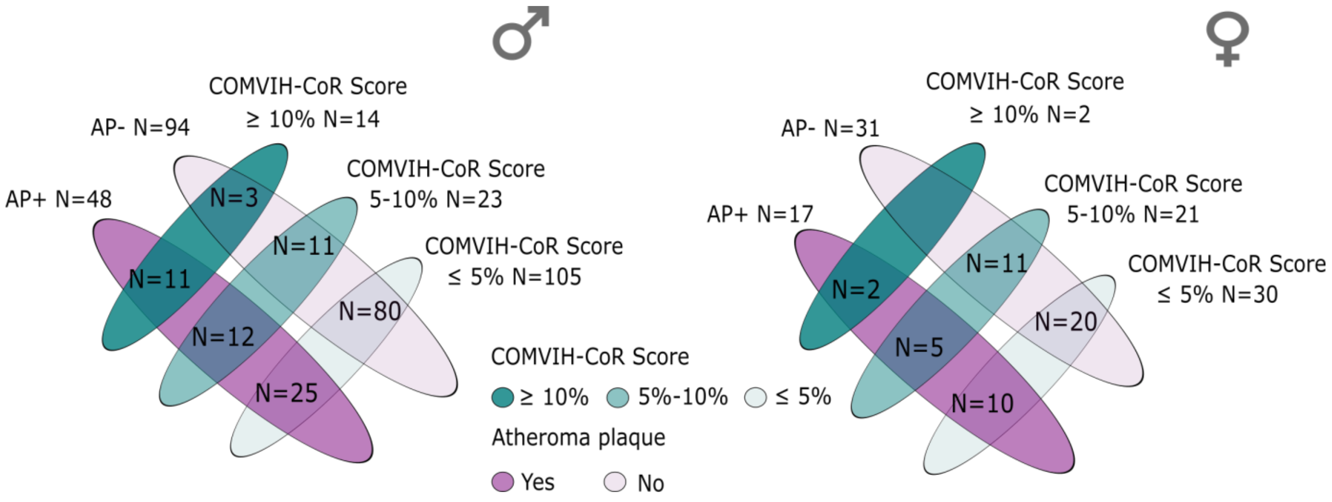


**Figure S3. Overlap between atheroma plaque status and cardiovascular risk categories stratified by sex in the HUMT cohort.**
Venn diagrams showing overlap of participants according to atheroma plaque presence/absence and COMVIH-CoR score categories (≤5% and ≥10%) in men (left) and women (right). Each region displays the number of individuals meeting the corresponding combination of criteria. Colors represent atheroma plaque status (purple = AP+, light purple = AP–) and COMVIH-CoR score categories (teal = ≥10%, light teal = ≤5%).
